# Predicting Affective Episodes in Bipolar Disorder Using Statistical Process Control Analysis of GPS-Based Mobility Patterns: Quantitative Study

**DOI:** 10.2196/77272

**Published:** 2026-06-22

**Authors:** Marvin Guth, Carl Bittendorf, Clemens Krug, Vera Miriam Ludwig, Esther Muehlbauer, Lisa-Marie Hartnagel, Emanuel Severus, Michael Bauer, Philipp Ritter, Ulrich W Ebner-Priemer

**Affiliations:** 1 Department of eHealth and Sports Analytics, Faculty of Sport Science, Ruhr University Bochum, Bochum, Germany; 2 Mental mHealth Lab, Institute of Sports and Sports Science, Karlsruhe Institute of Technology, Hertzstrasse 16, Karlsruhe, Baden-Württemberg, 76187, Germany, 49 72160844600; 3 Department of Psychiatry and Psychotherapy, University Hospital Carl Gustav Carus, Technische Universität Dresden, Dresden, Germany; 4 Asklepios Klinik Nord-Ochsenzoll, Hamburg, Germany; 5 Department of Psychiatry and Psychotherapy, Central Institute of Mental Health, Heidelberg University, Heidelberg, Germany

**Keywords:** bipolar disorder, mobile sensing, spatial data, spatial analysis, statistical process control, digital phenotyping, unique places

## Abstract

**Background:**

Bipolar disorders (BDs) represent a significant global health challenge, with frequent and severe affective episodes that impair quality of life. Accurate, early prediction of these episodes remains difficult. Recent advances in mobile sensing offer new possibilities to detect prodromal changes via smart digital phenotypes, such as geolocation data.

**Objective:**

This study aimed to examine whether spatial exploratory behavior, assessed via passive GPS data, can predict depressive and manic episodes in individuals with BD. Specifically, we evaluated the predictive value of unique places visited and related mobility metrics using statistical process control (SPC) techniques to identify both early deviations indicative of prodromal states and changes occurring during ongoing affective episodes.

**Methods:**

Using high-resolution GPS data from the BipoSense dataset, we applied Density-Based Spatial Clustering of Applications with Noise to extract behavioral mobility indicators: number of unique places visited, frequency of location changes, and time spent per location. We implemented exponentially weighted moving average (EWMA)–based SPC to identify “out-of-bounds” deviations from individual baselines. We then tested the alignment of these deviations with affective episodes and the prodromal periods. Optimization of SPC parameters (λ and control limit L) was performed to enhance predictive accuracy.

**Results:**

The analysis included 28 participants with BD and a total of 10,213 observation days, covering 26 depressive and 20 (hypo)manic episodes. Examining whether control limits distinguish affective episodes from euthymic days via multilevel models revealed that median time spent at clusters indicated both depressive and (hypo)manic episodes the best, whereas the number of unique clusters showed no significant association with phase transitions. While EWMA-SPC detected behavioral deviations during affective episodes, no single variable consistently met predefined thresholds for both sensitivity and specificity. Optimized SPC settings improved performance, but the number of unique places alone did not robustly predict prodromal or acute episodes. No statistically significant predictive accuracy (eg, sensitivity >70% and specificity >70%) was achieved for any individual indicator (*P*>.05). However, some SPC charts suggested within-person temporal deviations preceding episodes, indicating limited yet potentially informative patterns.

**Conclusions:**

Although unique places visited alone may not suffice as a predictive marker, the application of EWMA-based SPC to GPS data holds promise for the development of smart digital phenotypes. Although our analysis to predict upcoming episodes did not yield robust predictive accuracy in its current form, it provides a promising conceptual framework for individualized, low-burden behavioral monitoring. Further research is needed to refine existing digital biomarkers, develop new ones, and validate their clinical utility in reducing the frequency and severity of illness phases.

## Introduction

### Smart Digital Phenotyping in Bipolar Disorder

Bipolar disorders (BDs) affect up to 40 million people worldwide and represent a significant global burden of disease [1], imposing considerable personal and societal challenges [2]. Characterized by recurring episodes of depression and mania, the aim of BD disease management is to reduce the frequency, duration, or intensity of affective episodes, thereby extending the time spent in remission (euthymia) [3]. One promising strategy for maintaining euthymia is the early detection of emerging episodes, which facilitates timely interventions that may prevent the development of full-blown episodes. This can be achieved through intensive longitudinal study designs, such as digital phenotyping [4] and ambulatory assessment [5], as they enable continuous passive monitoring of symptom-related parameters over extended periods, thereby facilitating real-time feedback and automated forecasting.

Despite their potential, digital phenotyping approaches have yet to demonstrate a meaningful impact on disease trajectories in BD [6-10]. To address this, the need for clinically relevant smartphone-derived indices, so-called “smart digital phenotypes,” has been emphasized [11]. Unlike conventional smartphone-based measures, such as the number of incoming calls or steps per minute, smart digital phenotyping aims to extract clinically meaningful features that more closely reflect psychopathology. For instance, rather than measuring the number of incoming calls, analyzing speech patterns (eg, number of words spoken per minute [11]) may better capture the *Diagnostic and Statistical Manual of Mental Disorders* (Fifth Edition; *DSM-5*) criteria, such as talkativeness or pressure to keep talking [12]. Similarly, analyzing mobility patterns, such as visits to novel or unusual locations, may provide a more nuanced understanding of behavioral changes, compared to simply calculating daily kilometers traveled.

In particular, enhancing the analysis of location-based passive sensing data holds significant yet underused potential [13]. Given the rich behavioral insights embedded in raw GPS data, sophisticated geospatial analyses could substantially improve passive sensing approaches for BD, offering a novel and promising avenue for research.

### Previous Work

A recent systematic review of geospatial analyses in depression has shown multiple correlations across several key mobility metrics, such as entropy or distance. Notably, among 31 studies, 16 explored the relationship between depression and individual location-based data, 12 focused on predicting depression states, and 3 integrated location data with additional sensing modalities to assess depression [14]. The authors of the systematic review state that location data reflect changes in individual mood states and consistently exhibit a strong correlation with depression. Additionally, the reviewed studies demonstrate the predictive power and utility of location data in forecasting depressive symptoms.

However, the applicability of these study designs to BD is far from trivial. First and foremost, studies must not only ensure a sufficient number of participants to guarantee statistical power but also cover a sufficiently long observation period. Shorter study durations may fail to encapsulate critical episodes, thereby undermining the true potential of digital phenotyping [4]. Second, studies should involve individuals with a verified BD diagnosis, ideally reviewed by a clinician to ensure accurate ground truth, which is crucial for precise episode onset identification in analyses. Thus, precise identification of episode onset at the day level is critical. Even a deviation of only 3 days in a retrospective assessment spanning 3 months can blur the distinction between true prediction and the mere confirmation of an imprecisely recorded episode onset, especially when forecasts only anticipate episodes by a few days and lack a high-frequency, valid ground truth measurement [15]. Thus, several studies on digital phenotyping for BD episode prediction have yielded inconclusive results due to these challenges, as well as heterogeneous protocols and variable parameters [7].

Considering this, there are 2 studies worth mentioning that analyze spatial data to detect episodes in BD. Palmius et al [16] analyzed geographic data from 22 participants with BD and 14 healthy controls (HC), detecting depressive symptoms as measured via QIDS-SR16 (Quick Inventory of Depressive Symptomatology–Self-Report, 16 items) with 85% accuracy using location recordings alone. However, location variance was operationalized as “entropy” by clustering stationary locations via k-means, adjusting k until clusters were 400 m apart. This differs from classical spatial entropy in geography [17] and may limit the clinical relevance of smart digital phenotyping. A more clinically meaningful approach should distinguish between unique places visited, frequency of location changes, and time spent at each cluster. Additionally, the study’s short 3-month duration, limited symptom variance, and reliance on self-reported symptoms without expert classification pose significant limitations. Another relevant study examined differences in passively collected smartphone-based location data between 48 patients with BD and 31 HC [18], as well as investigating location data across different affective states for up to 9 months. Lower mobility was observed during depressive states compared to euthymic periods in patients with BD. Furthermore, patients with BD demonstrated lower location variance during affective states compared to euthymic periods. Limitations of this study, similar to those reported earlier, primarily concern the conceptualization of entropy, which poses challenges in constructing clinically relevant indices, as well as the study duration, which, although extended to up to 9 months, remains relatively short.

In sum, empirical findings on BD episode prediction from geospatial parameters remain inconclusive. This may be largely due to the methodological limitations mentioned earlier and the suboptimal operationalization of entropy. Most importantly, however, both studies adopt a dimensional approach to BD symptomatology, rather than distinguishing distinct affective episodes. This is evident in their reliance on self-reported symptom fluctuations, rather than focusing on the prediction of actual emerging episodes. However, this distinction is crucial because accurately predicting new affective episodes is essential for mitigating patient suffering and improving clinical care. Addressing these gaps requires well-designed longitudinal studies that capture a sufficient number of emerging episodes and accurately detect their onset [8].

### Spatial Movement Patterns in BD

Investigating the predictive properties of nuanced location-based passive sensing parameters in a sufficiently powered, temporally exact dataset to assess their clinical value in patients with BD holds significant promise. Changes in activity and energy are cardinal symptoms of BD: mania is, among other things, characterized by increased goal-directed activity and psychomotor agitation, whereas depressive episodes show reduced drive, diminished activity, and low energy (*DSM-5* [12]). These symptoms manifest in observable behavioral changes and translate to spatial movement patterns or exploratory behavior.

Empirical evidence supports this link. Animal models reveal altered exploratory behavior in mania-like [19] and depression-like [20] states. Reverse-translational models using an adapted version of the open field test (a standard rodent paradigm that assesses locomotor activity, anxiety-related behavior, and exploration) indicate that patients with BD display increased motor activity, more unpredictable exploration patterns, and more object interactions compared to HCs or patients with schizophrenia [21-23]. Notably, heightened activity and exploratory behavior persist even during euthymic phases [21,24,25]. Furthermore, it is generally assumed that individual movement patterns tend to be highly regular and predictable over time [26], as evidenced, for instance, by rather stable numbers of visited locations [27]. This raises an important question: Do patients with BD exhibit generally altered yet stable movement patterns as a trait, or do these patterns fluctuate according to symptomatology and affective state (eg, depressed, manic, and euthymic)?

To assess their clinical utility to predict and detect affective episodes, temporal dynamics of movement patterns in BD must be examined in detail. Current metrics, such as entropy used in the studies mentioned earlier, show routine but often fail to capture the full complexity of spatial behavior. It is crucial to differentiate between the time spent at a given location and the number of unique locations visited. For instance, very simplified, a regular daily commute might reflect euthymia, while frequent visits to different bars could indicate mania, whereas predominantly staying at home might signal depression. Understanding the timing and nature of these changes is key for early detection of emerging episodes.

These movement patterns can be effectively analyzed in spatial science cluster analysis tailored for longitudinal GPS data, combined with Geographic Information System–based mapping approaches, can visualize movement paths, hotspots, and clusters, and refine statistical parameters for greater accuracy. This simplifies complex data, uncovers hidden geographical patterns, and helps detect outliers, while also accommodating environmental factors such as geography, infrastructure, climate, and social context. Ultimately, advanced geospatial analysis of GPS data could be a valuable tool in developing smart digital phenotypes for BD.

### Statistical Process Control

Predicting emerging episodes relies not only on valid smartphone-derived indices but also on a prediction model. The exponentially weighted moving average (EWMA)–based statistical process control (SPC) method may offer a valuable approach in this context. SPC was originally developed to monitor industrial production processes and detect when the quality or performance of a product deviates significantly from expected standards by giving “out-of-bounds” indications [28]. This concept can also be applied to psychopathology, where euthymia is defined as a stable, functioning phase, and affective episodes are seen as processes that are “out of bounds” [29].

For instance, Schreuder et al [30,31] applied EWMA-SPC to repeated emotion assessments in at-risk and remitted adults, showing that deviations beyond control limits could signal persistent mental health problems or predict depressive episode recurrence. While SPC provided useful warning signals, its sensitivity remained limited, and its classification accuracy was moderate, highlighting both the promise and current limitations of the method [31].

EWMA-based SPC methods that use passive sensing and e-diary parameters have recently been used to detect prodromal symptoms of emerging affective episodes in patients with BD by Ludwig et al [15]. They tested whether digital phenotypes derived from passive sensing are marked as “out of bounds” during affective episodes and investigate whether these changes were already visible in the prodromal phases of affective episodes. Their results showed that, despite its potential as a low-burden tool, passive sensing did not reliably detect episodes or prodromal states. Self-reported bipolar mood was more effective than passive sensing in predicting current episodes, while prodromal phase prediction remained challenging. SPC with personalized control limits did not outperform established clinical cutoff scores, and optimizing SPC settings did not improve the balance between emerging episodes and false alarms. Ludwig et al [15] suggest that future research should focus on mobile sensing parameters more aligned with psychopathology to improve validity and sensitivity.

### Research Questions

Given the reported findings of altered exploratory behavior of humans and animals in mania-like and depression-like episodes, we investigated spatial movement patterns in patients with BD. To enable a comparison between episodes (euthymic, manic prodromal, manic, depressive prodromal, and depressive), we leveraged GPS data from the BipoSense dataset to analyze characteristics of the unique places visited by patients with BD (number of unique places visited, frequency of location changes, and time spent at each place). Although this study used the same dataset as Ludwig et al [15,32], our analytical approach differed.

The previous studies from this dataset focused on e-diary measures and on standard mobile sensing parameters. As the latter approach was disappointing, we argued that future studies might focus on mobile sensing parameters that more closely align with psychopathology. Developing smarter digital phenotypes, such as specific geolocation measures derived from GPS data, represent a promising direction, as these parameters can be aligned with empirical evidence, partly informed by animal models, while at the same time offering a low-burden, continuously monitored approach with substantial potential. Although Ludwig et al demonstrated that e-diaries still outperform passive sensing, passive sensing offers considerable advantages, as mentioned earlier. Consequently, our study aimed to identify a passive sensing parameter (or “smart digital phenotype”) that is most suitable for predicting emerging episodes, which is of highest priority from a clinical perspective.

To achieve this, we used EWMA-based SPC methods as a prediction model. We hypothesized that during depressive episodes, the EWMA-based number of unique places visited is lower (oversimplified: “fewer unique locations are visited”) and that the EWMA-based number of cluster changes in general is lower (oversimplified: “fewer changes between locations are happening”). Additionally, we hypothesized that the EWMA-based time spent at each place is higher (oversimplified: “individuals remain at specific locations longer”). Conversely, we expected the opposite pattern to emerge during manic episodes. Furthermore, we extended our investigation to prodromal phases, examining whether these same spatial behavior patterns precede the onset of depressive or (hypo)manic episodes. In fact, we hypothesized that the same EWMA-based spatial behavior patterns observed during depressive and (hypo)manic episodes will also manifest in prodromal phases, preceding full episodes and potentially allowing early detection. Second, we investigated whether the predictive accuracy can be enhanced by adjusting SPC settings (λ and L) and evaluate the resulting sensitivity and specificity in the context of potential clinical application.

## Methods

### Sample

To investigate our stated research questions, we used the BipoSense dataset [15,30,31], which offers continuous passive and active sensing over 12 months alongside biweekly expert interviews (26 interviews in total). Patients were recruited from a specialized outpatient clinic at Dresden University Hospital, meeting strict inclusion criteria, including a diagnosis of BD type I or II and a history of at least three affective episodes in the past 5 years [32,33]. A trained clinical psychologist conducted biweekly interviews (alternating between in-person and telephone) to assess categorical data on affective status (eg, being in a depressive episode, (hypo)manic episode, or euthymic) via the SCID-I section A (*DSM-5* criteria [34]) covering the previous 14 days. This served as “ground truth.” Moreover, dimensional symptom scores were assessed via standardized questionnaires, including Young Mania Rating Scale [35], the Bech-Rafaelsen Mania Rating Scale [36], and the Montgomery-Asberg Depression Rating Scale [37]. These tools covered symptoms over the preceding 3 days and demonstrated excellent reliability and validity. On the basis of the biweekly SCID interviews, day-level mood status (depressed, euthymic, and (hypo)manic) was labeled for the previous 14 days. Each mood episode contributed to multiple analytical phases: preepisode weeks were categorized as early prodromal (days −14 to −8) and late prodromal (days −7 to −1), following previous research [15,32,38], and stratified by episode polarity. The onset and subsequent course were further classified as first week (days 1‐7), second week (days 8‐14), and ongoing (day 15 onward). This structure enabled the identification of temporal dynamics surrounding episode onset.

Of the 112 patients who initially contacted the BipoSense study, 53 (47.3%) agreed to undergo screening for eligibility. Ultimately, 31 (27.7%) patients were enrolled in the study. Reasons for exclusion included not meeting the eligibility criteria (n=15, 13.4%), nonresponse after the initial contact (n=25, 22.3%), living too far from the study site (n=18, 16.1%), and technical concerns (n=4, 3.6%), while others did not provide reasons for refusal [33].

### Measures

Digital phenotyping was conducted via the app movisensXS [39] (movisens GmbH, Karlsruhe, Germany). In addition to standard e-diary variables, such as end-of-day entries on sleep (time spent asleep, awake, or sleepless in bed over the previous 24 hours, recorded in 60-min segments) and medication use (both adapted from ChronoRecord, a rigorously validated electronic mood charting system [40,41]), a wide range of passive sensing variables was collected. In this study, we focused on indices of unique places as derived from GPS location data.

### Data Preparation

**Figure 1.**
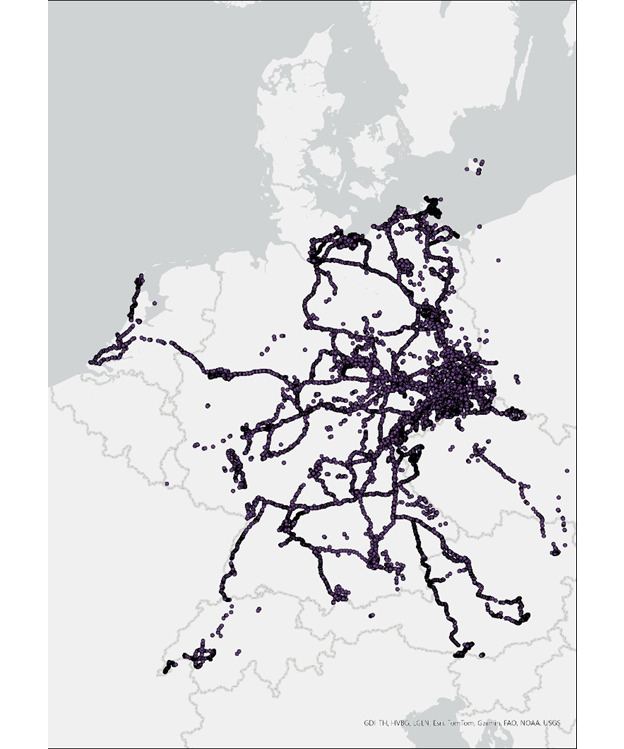
Spatial distribution of all GPS points (14,483,536 entries) of all participants (n=28). Owing to the fact that the study took place at University Hospital Dresden, the majority of the points are concentrated in and around Dresden.

Initially, 31 participants were included. After 3 weeks, 1 (3.2%) participant dropped out, leaving 30 (96.8%) with at least 10 months of data. One participant was excluded due to data extraction issues, and another was excluded because of the absence of 6 euthymic weeks. The final sample comprised 28 participants. For this sample, geospatial data were available at a very high resolution of approximately 5 to 10 seconds, totaling 8,616,671 data points. To optimize computational efficiency, the data were downsampled to 1 entry per minute, resulting in a reduced dataset comprising 1,861,321 data points. Afterward, the Haversine algorithm was used to calculate distances between consecutive entries. To ensure data quality, entries were filtered out if the distance from the previous point implied an exceeded speed of 300 km/h, as such deviations may result from inaccurate GPS signals, often due to battery-saving measures using GSM data instead of GPS satellites. To make a cluster analysis feasible, the points were projected from the global geodetic coordinate system, WGS84 (EPSG 4326) to UTM 32 (EPSG 25832). Owing to energy efficiency, GPS data collected via movisensXS were not recorded continuously, causing temporal gaps. If a participant remains stationary, new GPS coordinates were only captured when a change in location occurs, evidenced by a signal in the acceleration sensor of the smartphone. Accordingly, missing entries were filled with the value of the last nonmissing entry, leading to a dataset containing 14,483,536 points. The spatial distribution of all GPS points of all participants is presented in Figure 1.

### DBSCAN Cluster Analysis

To identify unique places, the density-based spatial clustering algorithm DBSCAN (Density-Based Spatial Clustering of Applications with Noise [42]) was used. Unlike k-means, DBSCAN does not require a predefined number of clusters and classifies observations as core points, border points, or noise points. Its nonparametric density estimation allows for the detection of complex cluster shapes, making it particularly effective for data with varying densities and irregular structures such as spatial data [40]. On the basis of visual tests of BipoSense data, we set epsilon to 100 m and n to 20 minutes per day. In practical terms, core points must fall within a 100 m radius to form a cluster, requiring at least 20 points.

The DBSCAN algorithm considers all points within the predefined radius (epsilon). To prevent frequently traveled routes where GPS points overlap from being defined as clusters, an additional criterion was applied: a cluster must involve a minimum of 10 consecutive minutes spent at a unique place. Thus, a cluster is defined as “a minimum of 20 GPS measurements or minutes that fall within a 100-m diameter with at least 10 consecutive minutes spent at that location.”

On the basis of the spatial data and the conducted DBSCAN, we derived the following variables:

- UniqueClusters: the number of unique locations visited by the participant on that day
- ClusterChanges: the number of location (cluster) changes, grouped by each participant and each day
- MedianTimeAtCluster: the median number of minutes spent across all clusters, grouped by each participant and each day

### Statistical Process Control

The central assumption was that (upcoming) depressive or manic episodes are associated with changes in behavior, which in turn are reflected in changes in the means and/or variances of appropriate mobile sensing parameters compared to a 28-day baseline period. To ensure that this baseline period reflected a stable euthymic state, it was only assigned when biweekly clinician ratings indicated the absence of an affective episode. If a participant experienced an episode at study entry, baseline assignment was postponed until a confirmed euthymic phase. Additionally, we compared mean self-rated and clinician-rated mood scores between the baseline and all other phase categories. We attempted to detect these shifts using EWMA-based control charts, which smooth the incoming data using an exponentially weighted moving average of previous values, minimizing the influence of noise. If the EWMA value exceeds or falls below control limits established using the mean and variance of the baseline period, this should be an indicator of an episode.

**Figure 2.**
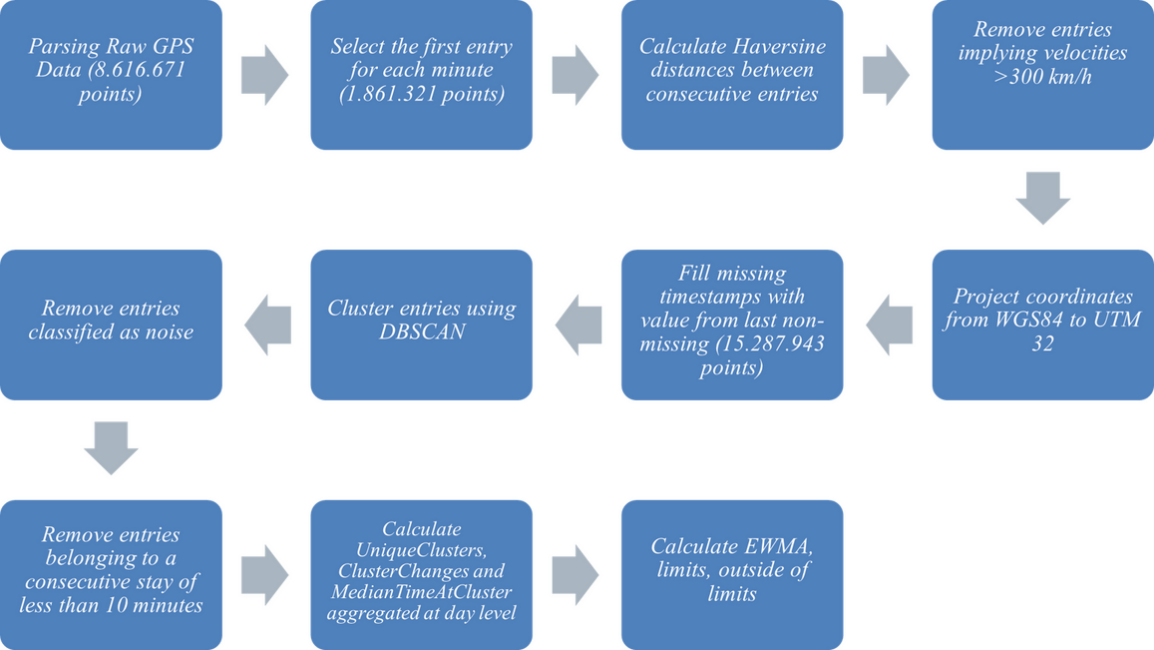
Overview of the GPS data processing methodology. The initial raw GPS data are refined through a sequence of procedures encompassing data parsing, coordinate transformation (WGS84 to UTM), Density-Based Spatial Clustering of Applications with Noise (DBSCAN) clustering, and exponentially weighted moving average (EWMA) calculation.

There are 2 parameters that can be used to tune the sensitivity and specificity of the method: λ and average run length (ARL₀). The smoothing parameter λ determines how much weight is given to the value of the most recent observation. The closer λ is to 0, the more weight is given to the previous days. Conversely, the closer λ is to 1, the smaller the smoothing effect and the more sensitive the EWMA is to changes. The ARL₀ is the expected number of days before a false-positive alert occurs; that is, the limits are exceeded or undercut although there was no underlying change. λ and ARL₀ were used to calculate another parameter L, which determines the width of the control limits. We set λ to 0.15 and decided on an ARL₀ of 180 days, which resulted in an L of 2.536435. For comparability and methodological consistency, these parameters were applied globally across participants rather than optimized individually. This approach was chosen based on prior SPC research in affective disorders [15,29-31]. We provide a detailed illustration of SPC charts with varying parameters λ and L using synthetic data to demonstrate the principles of EWMA-based SPC (see Figures S1 and S2 in MultimediaAppendices 1  2). Additionally, a detailed tutorial on the application of the EWMA procedure for detecting real-time changes in intensive longitudinal psychological data is provided by Smit et al [29]. A complete list of all data processing steps is shown in Figure 2.

### Generalized Linear Mixed Models

To account for the nested structure of daily observations within participants, multilevel (mixed effects) models were estimated, incorporating both fixed and random effects. Specifically, multilevel logistic regression models were applied. Each unique place parameter was treated as the independent variable (fixed main effect), while disorder status served as the binary-dependent variable. For the models, unique place parameters were dichotomized to reflect whether daily values were within or outside the control limits. To address the risk of α-error inflation due to multiple comparisons (3 variables tested across 9 disorder status contrasts), a Bonferroni-Holm correction was applied separately for the depressive and manic states, reflecting the differing number of phases. Thus, for depression, the smallest *P* value was multiplied by 15 (3×5), the second smallest by 14, and so forth. For mania, the correction followed the same scheme, starting with a multiplication factor of 12 (3×4). Upon reasonable request, day-level data will be provided.

### Sensitivity and Specificity

To better assess the potential clinical value of an SPC-based early warning system, we evaluated its sensitivity and specificity. Sensitivity represents the proportion of affective episodes correctly detected out of all episodes, while specificity reflects the proportion of euthymic days accurately classified out of all euthymic days. In clinical practice, balancing sensitivity and specificity is essential. While high sensitivity is necessary to capture most emerging episodes, an excessive number of false positives may reduce the likelihood that patients and clinicians respond to frequent alerts. We used established cutoffs for both sensitivity (50%) and specificity (95%) [15] translating into detecting at least 50% of new emerging episodes while having less than one false alarm per month.

### Ethical Considerations

The study adhered to the Declaration of Helsinki and was approved by the ethics committee at the Technical University of Dresden (EK-Nr.: 26012014). All participants provided written informed consent prior to participating in the study. After having signed informed consent, patients received a study smartphone (optional) and reimbursement of €35 (US $40.7) per month [33]. All self-report data were pseudonymized, and personal identifiers were removed prior to analysis. Raw GPS data, which could potentially allow for the reidentification of individual participants, are not publicly shared and were strictly accessible only to the research team, thereby ensuring the protection of participant privacy.

## Results

### Sample Characteristics

As described previously, 28 participants were included in our analyses, with a mean age of 43.82 (SD 12.09) years, ranging from 25 to 70 years. The cohort consisted of 16 (57.1%) female and 12 (42.9%) male participants, of whom 16 (57.1%) were diagnosed with BD type I and 12 (42.9%) with BD type II. Participant compliance in the BipoSense dataset was notably high. In total, 726 (97%) of 749 biweekly diagnostic visits were completed. Participants completed 9433 (89%) evening e-diary entries, with 1154 (11%) entries missing due to technical issues, low battery, or nonresponse [33]. Over the course of the assessment period, mobile sensing data were recorded for 10,213 (99%) patient days, covering 26 depressive and 20 (hypo)manic episodes. This included 7851 (76.9%) euthymic days, 161 (1.5%) days classified as early prodromal depressive, 164 (1.6%) days classified as late prodromal depressive, 179 (1.8%) days marking the onset of a depressive episode, 168 (1.6%) days within a second depressive episode, and 194 (1.9%) days categorized as part of ongoing depressive phases. In total, 866 days (8.5% of all observation days) were classified as depressive phases across all participants. Regarding (hypo)mania, the dataset comprised 133 (1.3%) early prodromal days, 141 (1.4%) late prodromal days, 147 (1.4%) days marking the onset of a (hypo)manic episode, 146 (1.4%) days within a second (hypo)manic episode, and 98 (1.0%) days of ongoing (hypo)manic states. This resulted in 665 days (6.5% of all observation days) classified as manic episodes. Additionally, 856 (8.4%) days were marked as missing. For statistical analysis, extended (hypo)manic episodes lasting longer than 3 weeks were excluded due to an insufficient number of recorded days.

### EWMA-Based SPC Charts

**Figure 3.**
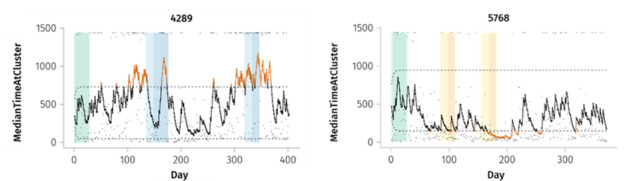
Exponentially weighted moving average (EWMA) statistical process control chart for participants 4289 and 5768 for the variable MedianTimeAtCluster. The x-axis represents the days of the study, and the y-axis shows the median time participants spent at a cluster. The baseline period used to compute personalized upper and lower control limits is depicted by the green area. Yellow areas indicate (hypo)manic episodes, and blue areas indicate depressive episodes. Shaded regions mark the 2 prodromal weeks (early and late prodromal). Black dots represent daily raw values, and the black line depicts the EWMA. The line turns red when the EWMA exceeds the control limits.

Figure 3 shows 2 example SPC plots for the MedianTimeAtCluster variable in 2 patients, that is, 4289 and 5768. The baseline phase is depicted by the green area, while affective episodes are color-coded (yellow for manic episodes and blue for depressive episodes). Prodromal phases are denoted by lighter shaded areas that precede the affective episodes. The daily EWMA is depicted in black when within the upper and lower control limits and in red when values exceed these thresholds. To verify that the baseline periods represented stable mood states suitable for EWMA phase I initialization, we compared clinician-rated and self-rated symptom scores across all phase categories. Ensuring that the baseline represents a stable state is crucial for the validity of the EWMA procedure. As expected, baseline scores closely resembled those observed during euthymia, with minimal symptom burden (eg, baseline YMRS: mean 1.06, SD 1.95 vs euthymia YMRS: mean 1.06, SD 1.88; baseline self-rating: mean 49.25, SD 12.45 vs euthymia: mean 49.88, SD 10.46). In contrast, both manic and depressive phases were clearly distinguishable from baseline. For example, during depressive phases, participants showed low YMRS scores (first week: mean 0.54, SD 1.24; ongoing: mean 0.61, SD 1.08) alongside reduced self-rated mood (first week: mean 39.71, SD 14.60; ongoing: mean 31.14, SD 16.31), whereas during manic phases, YMRS increased (first week: mean 9.57, SD 5.07; ongoing: mean 9.98, SD 4.42) with correspondingly higher self-rated mood (first week: mean 54.88, SD 9.37; ongoing: mean 51.24, SD 10.40). These differences confirm that baseline periods were indeed “in control” and distinctly separate from symptomatic phases. A complete overview of clinician-rated and self-rated symptom scores across all phase categories is provided in Table S1 in Multimedia Appendix 3.

As can be seen, the EWMA-based median time per cluster significantly fluctuates over time and shows low values during manic episodes, whereas values are higher during and after a depressive episode. Nevertheless, we have to strongly emphasize that we selected those particular exemplar figures because they illustrate the proposed concept best. A more comprehensive examination of all variables across all patients revealed a more ambiguous picture. SPC charts for all observed parameters from patients 4289 and 5768 can be found in Figure S3 in Multimedia Appendix 4.

### Testing the SPC Assumptions on Unique Places Visited

#### Overview

Table 1 presents the results of the multilevel models examining whether control limits distinguish affective episodes from euthymic days. In reference to Figure 3, these models assess whether the proportion of “red dots” is higher during affective episodes compared to euthymic periods.

**Table 1.** Estimates from multilevel logit models differentiating disorder status^a^.

Disorder status and phase	Unique clusters		Cluster changes		Median time at cluster	
	OR^b^ (95% CI)	*P* value	OR (95% CI)	*P* value	OR (95% CI)	*P* value
Depressive episodes versus euthymia						
Early prodromal	0.623 (0.34-1.15)	.13	0.920 (0.86-0.98)	.01	1.002 (1.00-1.004)	.01
Late prodromal	0.803 (0.56-1.16)	.24	0.978 (0.96-0.99)	.01	1.001 (0.99-1.01)	.82
1st week depressive episode	0.910 (0.81-1.03)	.12	1.007 (1.00-1.02)	.08	1.002 (1.00-1.01)	.39
2nd week depressive episode	0.826 (0.14-4.73)	.83	1.017 (0.82-1-27)	.88	1.001 (1.00-1.002)	.08
Ongoing depressive weeks	0.803 (0.58-1.12)	.19	0.975 (0.94-1.02)	.17	1.001 (1.00-1.002)	<.001
(Hypo)manic episodes versus euthymia						
Early prodromal	0.997 (0.99-1.01)	.45	1.031 (0.91-1.17)	.63	1.001 (1.00-1.002)	.05
Late prodromal	0.782 (0.60-1.02)	.07	0.985 (0.97-1.01)	.12	1.002 (1.00-1.01)	.56
1st week (hypo)manic episode	0.958 (0.46-2.02)	.91	1.012 (0.96-1.05)	.53	1.001 (1.00-1.002)	<.001
2nd week (hypo)manic episode	1.128 (0.83-1.53)	.44	1.031 (1.01-1.06)	.01	1.000 (1.00-1.001)	.05

a Disorder status is categorized into the following phases: “early prodromal” (days 14 to 8 prior to episode onset), “late prodromal” (days 7 to 1 before onset), “first week” (days 1 to 7 of the episode), “second week” (days 8 to 14), and “ongoing weeks” (day 15 onward), with separate classifications for (hypo)mania and depression. “Euthymia” serves as the reference category.

b OR: odds ratio.

In the early prodromal phase preceding depressive episodes, a lower number of EWMA-based unique clusters (odds ratio [OR] 0.623, 95% CI 0.34-1.15) was observed, although this is not statistically significant. EWMA-based cluster changes (OR 0.920, 95% CI 0.86-0.98; *P*=.01) show a significant negative association, indicating fewer cluster transitions in this phase. EWMA-based median time spent at each cluster (OR 1.002, 95% CI 1.0-1.004; *P*=.01) is significantly elevated, suggesting that higher median time spent at clusters is linked to an increased likelihood of entering a depressive episode. During the late prodromal phase, EWMA-based unique clusters (OR 0.803, 95% CI 0.56-1.12), EWMA-based cluster changes (OR 0.978, 95% CI 0.96-1.0), and EWMA-based median cluster values (OR 1.001, 95% CI 0.9-1.01) show no statistically significant associations with depressive onset. During the first and second weeks of depressive episodes, no cluster indicators show significant effects. In ongoing depressive episodes, EWMA-based unique clusters (OR 0.803, 95% CI 0.58-1.1) and EWMA-based cluster changes (OR 0.975, 95% CI 0.96-1.05) are not significantly associated with the disorder status. However, EWMA-based median time spent at clusters (OR 1.001, 95% CI 1.000-1.001; *P*<.001) is significantly elevated.

Regarding (hypo)manic episodes, in the early prodromal phase as well as late prodromal phase, EWMA-based unique clusters, EWMA-based cluster changes, and EWMA-based median time spent values show no statistically significant associations. During the first week of a (hypo)manic episode, EWMA-based unique clusters (OR 0.958, 95% CI 0.46-2.02) and EWMA-based cluster changes (OR 1.012, 95% CI 0.97-1.05) are not significantly related to disorder status. However, EWMA-based median time spent at cluster (OR 1.001, 95% CI 1.000-1.002) demonstrated a small but statistically significant association with (hypo)manic symptom onset. In the second week of a (hypo)manic episode, EWMA-based unique clusters and EWMA-based MedianTimeAtCluster show no significant effects. In contrast, EWMA-based cluster changes (OR 1.031, 95% CI 1.01-1.06; *P*=.01) exhibit a significant positive association, indicating increased cluster variability during this phase.

#### Bonferroni-Holm Correction

To account for random findings caused by multiple testing, we used a Bonferroni-Holm correction, as described in the *Methods* section. From the reported 6 significant findings in Table 1, only 2 remained significant, namely, EWMA-based MedianTimeAtCluster for ongoing depressive weeks (*P*=.01) and EWMA-based MedianTimeAtCluster for the 1st week of (hypo)manic episode (*P*=.004).

### Optimizing Sensitivity and Specificity

**Figure 4.**
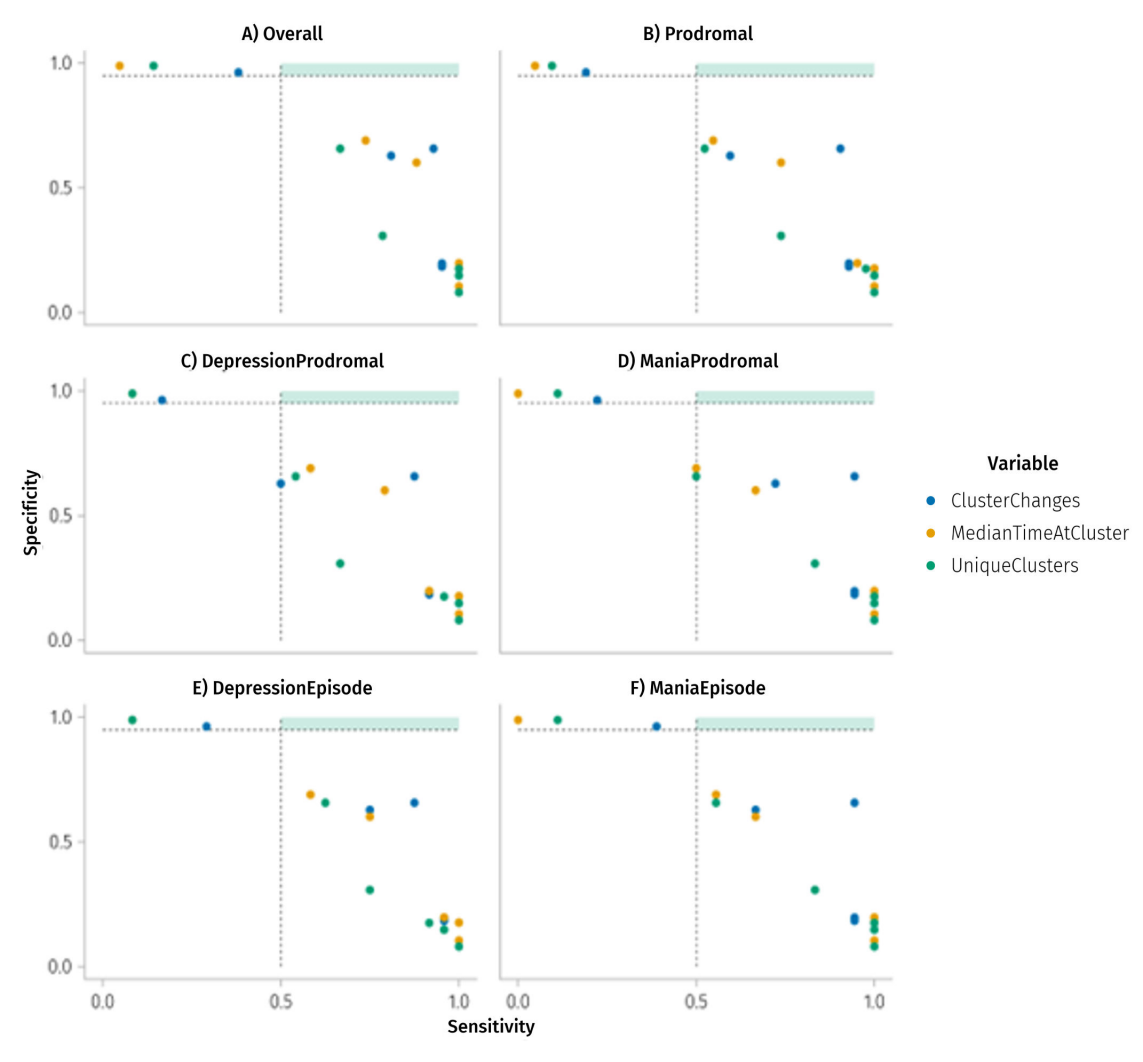
Results for sensitivity and specificity after optimizing the parameters λ and L are presented, divided according to different periods and clinical phases: (A) Overall represents the entire assessment period, (B) prodromal includes all prodromal days, (C) depression prodromal covers only depressive prodromal days, (D) mania prodromal covers only manic prodromal days, (E) depression includes all depressive days, and (F) mania includes only manic days during optimization. The dotted lines and the green area indicate the suggested threshold of 50% sensitivity and 95% specificity, respectively, which we consider a prerequisite for clinical application.

In the next step, the clinical applicability of an SPC-based early warning system was evaluated through the computation of sensitivity and specificity metrics. Before conducting the analysis, a threshold was defined whereby identifying at least every second impending episode was considered satisfactory (sensitivity >50%), while false-positive alerts were to remain limited to a maximum of one per month (specificity >95%). As detailed in the *Methods* section, the SPC parameters λ and L were systematically optimized and assessed in accordance with 6 distinct optimization criteria. Figure 4 compiles and graphically depicts the sensitivity and specificity evaluations. The green markers in the upper-right segment represent the predetermined cutoff thresholds. Cluster parameters are highlighted using color distinctions.

While some configurations approach the predefined thresholds of sensitivity (>50%) and specificity (>95%), none of the evaluated variables met both criteria at the same time. Specificity is often too low, particularly for detecting depressive and manic episodes (panels E and F), leading to excessive false positives. In contrast, prodromal and depression prodromal phases showed a better balance, suggesting potential clinical utility in these cases. The 3 variables (ClusterChanges, MedianTimeAtCluster, and UniqueClusters) vary in performance, without a clear best predictor. For a complete overview of the results, please refer to Table S2 in Multimedia Appendix 5.

## Discussion

### Principal Findings

This study investigated the application of EWMA-based SPC methods using spatial movement patterns to detect prodromal symptoms of emerging affective episodes in individuals with BD. Building upon previous work, we examined whether location-based smart digital phenotypes derived from GPS data, namely the number of unique places visited, the frequency of location (cluster) changes, and the median number of minutes spent across all clusters could effectively capture the dynamic changes in behavior associated with different phases of BD.

First, we observed that, at least in BD, individual movement patterns indeed vary over time, in contrast to the findings of González et al [26] and Allessandretti et al [27]. Regarding our hypotheses, visual descriptive approaches, such as examining SPC charts, demonstrated the potential of this method. During depressive phases, we observed that fewer locations were visited, and individuals tended to spend longer durations at specific locations, which aligns with our hypothesis. Conversely, during manic episodes, the opposite pattern emerged, which was consistent with our expectations. Notably, it is observed that the out-of-bounds processes were flagged even before the onset of the episodes, which may hold potential for prospective phase prediction and eventual clinical application. It can be assumed that during depressive episodes, individuals spend more time in a cluster, reflecting symptoms of apathy, whereas during manic episodes, they spend significantly less time in such clusters, indicating restlessness and increased mobility. This observation is in line with animal models that demonstrate altered exploratory behavior in mania-like [19] and depression-like [20] states.

However, a broader analysis across all patients and variables (Table 1) reveals a more complex and ambiguous picture. Examining whether control limits distinguish affective episodes from euthymic days via multilevel models revealed that overall, the median time spent at clusters emerged as the most consistent indicator across both depressive and (hypo)manic episodes. In contrast, the number of unique clusters was generally not significantly associated with phase transitions, even before Bonferroni-Holm correction. These patterns suggest that the frequency of location changes may serve as a more robust marker of affective episodes than the sheer number of distinct locations visited. Specifically, participants experiencing depressive episodes tend to remain temporally and spatially bound, whereas those in (hypo)manic episodes are highly mobile. In the latter group, individuals tend to move repeatedly between certain locations rather than visiting a large number of distinct places. Moreover, the phase-specific significance indicates that different aspects of movement behavior become relevant at different stages of episode development, highlighting the complexity of predicting mood episodes from passive sensing data.

Although our dataset is of high quality, featuring 1 year of longitudinal data and frequent reliable expert interviews, creating ideal conditions for applying SPC methods, digital phenotypes did not consistently prove reliable in detecting prodromal states and affective episodes through SPC analysis. Thus, the results partially support our initial hypotheses, suggesting that the analysis of visits to unique places may hold promise as a tool for capturing subtle behavioral changes indicative of impending mood shifts. In our descriptive investigation of optimizing predictions through systematic adjustments of SPC settings, we found that the inability to achieve sufficient sensitivity and specificity, even post hoc with the optimal parameter combinations, challenges the approach and/or the variables used. Previous EWMA-SPC studies on intensive emotion data indicated early warning signals for depressive episodes, but with limited sensitivity [31]. Consistent with this, our application to GPS-derived movement patterns showed some phase-specific behavioral changes, yet predictive accuracy remained insufficient, highlighting both the potential and current limitations of SPC for real-time episode detection.

Comparing our findings to previous studies, our results are in line with and expand upon the observations of Palmius et al [16] and Faurholt-Jepsen et al [18], who reported correlations between spatial movement patterns and mood states. While these earlier studies primarily focused on symptom correlations, our approach incorporates clearly defined episode onsets and aims to predict episodes prospectively. The more differentiated variables used here (unique clusters, cluster changes, and median time) allow for a more nuanced characterization of spatial behavior. This provides a more granular understanding of how changes in spatial behavior precede and accompany mood episodes, highlighting both consistencies and divergences with prior work. However, in direct comparison to Ludwig et al [15], self-rated mood appeared to outperform even the more advanced smart digital phenotypes examined in this study, although its ability to predict prodromal phases remains limited as well.

While systematic reviews of geospatial analyses in affective disorders [14] demonstrate remarkable potential for predicting depressive symptoms, their application to BD, as outlined earlier, is far from trivial. Moreover, predicting full episodes remains particularly challenging as demonstrated in our analysis. However, to effectively advance research on BD and integrate digital phenotyping into clinical practice, it is essential not only to adopt a purely dimensional approach to BD symptomatology [16,18] but also to differentiate between distinct illness phases and predict their occurrence. As demonstrated by Palmius et al [16] in the analysis of spatial patterns and Faurholt-Jepsen et al [18] in the analysis of unique places, while clear correlations were identified, the transfer to episode prediction remains challenging.

In summary, while the analysis of visits to unique places offers a low-burden, continuous monitoring approach with high potential, our findings did not confirm the consistent effectiveness of EWMA-based SPC methods for predicting affective episodes using passive sensing data in clinical practice. Furthermore, the use of EWMA-based SPC with individualized control limits did not surpass established threshold values.

### Limitations

First, although the study exceeded the duration of previous studies by a significant margin and included patients with a high number of prior episodes, providing the most temporally precise labeling of days per patient, the number of newly emerging episodes remained limited. Future research aimed at predicting new episodes may benefit from longer study durations, such as the 18-month period used in our most recent randomized controlled trial [5]. Second, while it has been suggested that frequent assessments of psychopathological status could prevent the occurrence of new episodes, our dataset provides no empirical evidence to support this claim [33]. Third, our study deviated from the intended chronological sequence in 7 cases due to insufficient initial euthymic days, requiring retrospective upper control limit or lower control limit application and compromising the SPC’s prospective nature. While this aimed to capture more affective episodes, it may have introduced confounding effects from postepisode behavioral changes, such as increased activity after depression. Moreover, participants were aware that their GPS data were being collected, which may have influenced their behavior due to a potential Hawthorne effect. However, as our study covered an extended observation period of 365 days, we do not assume that participants consistently altered their behavior throughout the entire duration. Another limitation concerns the evaluation of model performance, which was based on prespecified operating thresholds (≥95% specificity and ≥50% sensitivity) chosen based on clinical reasoning rather than empirically derived or standardized criteria. While this choice reflects the practical prioritization of minimizing false-positive alerts in real-world clinical settings, it necessarily introduces a degree of subjectivity into the performance assessment. In particular, the strict specificity constraint may limit achievable sensitivity and thereby influence the apparent trade-off between both metrics. Moreover, the absence of established guidelines for optimal threshold selection in SPC-based early warning systems for affective disorders limits the comparability of our results to other approaches and may affect generalizability across different clinical contexts and monitoring frequencies. Finally, prodromal phases were defined exclusively by a temporal criterion, specifically the 2-week period prior to affective episodes, without clinical assessment of actual prodromal symptoms. As a result, we lack a “ground truth” and cannot rule out the possibility that patients may not have exhibited any prodromal symptoms to capture to begin with, whether via passive sensing or self-reported bipolar mood. This observation is consistent with clinical reports in which patients sometimes describe a sudden “switch” from euthymia to an affective episode overnight. Our data may partially support this hypothesis, as, at least for (hypo)mania, late prodromal days were more frequently differentiated from euthymia than early prodromal days.

### Future Directions

Although our study makes a significant contribution to the current state-of-the-art research by developing smart digital phenotypes based on GPS data in patients with BD, we have only scratched the surface with regard to location-based passive sensing. First, we acknowledge that no direct comparison with classical entropy-based mobility metrics was conducted in this study. While our DBSCAN-based feature set was designed to capture more granular spatial-temporal structure than aggregated entropy measures, we cannot empirically claim superiority over entropy without a head-to-head evaluation. Importantly, from a theoretical standpoint for longitudinal spatial data, DBSCAN is particularly well suited, as it preserves discrete location states and transitions, which are otherwise compressed in entropy formulations. This property is essential for enabling the derivation and interpretation of the specific variables used in this work (UniqueClusters, ClusterChanges, and MedianTimeAtCluster), which explicitly depend on identifiable spatial clusters rather than aggregate distributional summaries. Future work should explicitly benchmark entropy-based and cluster-based mobility representations within the same predictive framework to quantify their relative clinical utility. Second, there are numerous other potential parameters that could correspond closely to psychopathological features. These include exposure to highly frequented areas, such as city centers and shopping malls, as well as exposure to green spaces or urban environments [43]. Additionally, interactions related to residential locations present a promising avenue for future exploration. Thus, future research should explore how multiple behavioral and physiological indicators can be integrated into a more comprehensive model. Furthermore, incorporating machine learning algorithms to identify complex patterns across multiple variables could improve the sensitivity and specificity of the model. Finally, regarding our SPC methods, previous work suggests that combining changes in variability (eg, SD) with mean-level changes may improve the sensitivity of early warning signals [31,44]. In our study, we focused on mean-based SPC indicators, which may have contributed to the limited sensitivity observed. Future research should therefore explore combined approaches integrating both mean and variability-based indicators.

## Conclusions

In conclusion, this study contributes to the growing body of evidence supporting the use of digital phenotyping in BD with a particular focus on location-based passive sensing, specifically visits to unique places by individuals with BD. While SPC charts highlight the potential of this approach, a broader analysis across all patients and variables reveals a more complex and ambiguous picture, with overall effects being limited and of insufficient clinical applicability. Notably, after applying Bonferroni-Holm corrections to our generalized mixed models, only time spent at each cluster remained of some significance for certain phases, underscoring the selective predictive value of this approach. The comparison between SPC charts and the statistical modeling approach showed that further research is needed to refine and validate these findings. Yet, our results underscore the conceptual value of digital phenotyping for improving our understanding and monitoring of BD. By leveraging the power of mobile technology and data analytics, we may move toward a future where personalized interventions could be delivered proactively.

## Supplementary material

10.2196/77272 Multimedia Appendix 1 Eexponentially weighted moving average charts illustrating the effect of varying control limits (L=2.0, 2.54, and 3.0), which correspond to different average run lengths (ARL0) and thus alter the sensitivity to process variation.

10.2196/77272 Multimedia Appendix 2 Exponentially weighted moving average charts illustrating the impact of different smoothing parameters (λ=0.05, 0.15, and 0.3) on the detection of shifts in synthetic process data.

10.2196/77272 Multimedia Appendix 3 Scores of clinician-rated and self-rated measures across baseline and the 11 mood phases.

10.2196/77272 Multimedia Appendix 4 Exponentially weighted moving average (EWMA) statistical process control (SPC) chart for participants 4289 and 5768 for all remaining variables of the study.

10.2196/77272 Multimedia Appendix 5 Results of the optimization procedure of λ and L.

## Abbreviations

ARL: average run length
BD: bipolar disorder
DBSCAN: Density-Based Spatial Clustering of Applications with Noise
*DSM-5*: *Diagnostic and Statistical Manual of Mental Disorders* (Fifth Edition)
EWMA: exponentially weighted moving average
HC: healthy control
OR: odds ratio
QIDS-SR16: Quick Inventory of Depressive Symptomatology–Self-Report, 16 items
SPC: statistical process control
