# Supplementary material for: Predicting Affective Episodes in Bipolar Disorder Using Statistical Process Control Analysis of GPS-Based Mobility Patterns: Quantitative Study
Source: JMIR Mhealth Uhealth. 2026 Jun 22;14:e77272. doi: 10.2196/77272 (PMC13286074; doi:10.2196/77272)
Supplement: Multimedia Appendix 1 [file mhealth-v14-e77272-s001.docx]

**
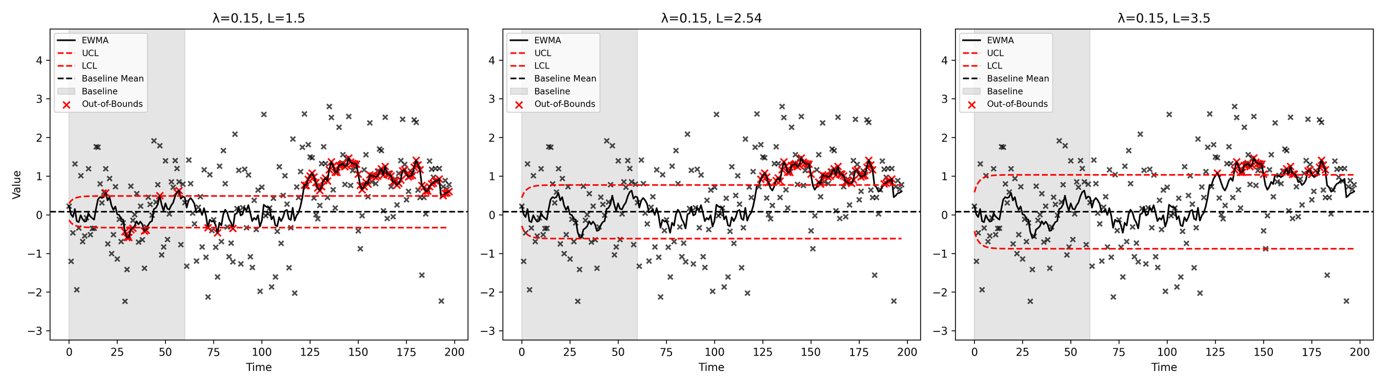
**

Supplementary Figure S 1: EWMA charts illustrating the effect of varying control limits (L = 2.0, 2.54, 3.0), which correspond to different average run lengths (ARL0) and thus alter the sensitivity to process variation. The baseline and plotting conventions are identical to the top panel. Narrower control limits (lower L) increase the number of out-of-bounds detections, while wider limits reduce false alarms, highlighting the trade-off between sensitivity and specificity in statistical process control.
