# Supplementary material for: Predicting Affective Episodes in Bipolar Disorder Using Statistical Process Control Analysis of GPS-Based Mobility Patterns: Quantitative Study
Source: JMIR Mhealth Uhealth. 2026 Jun 22;14:e77272. doi: 10.2196/77272 (PMC13286074; doi:10.2196/77272)
Supplement: Multimedia Appendix 2 [file mhealth-v14-e77272-s002.docx]

**
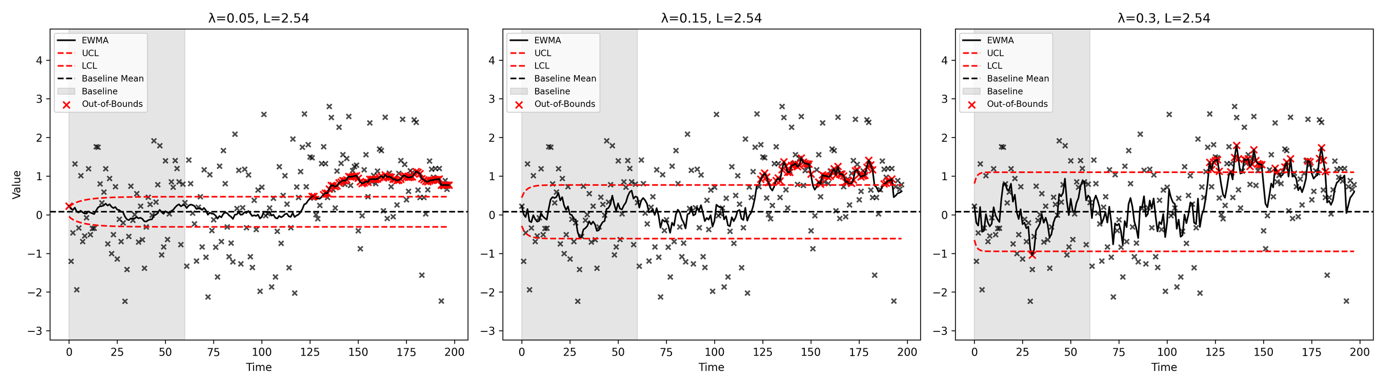
**

Supplementary Figure S 2: EWMA charts illustrating the impact of different smoothing parameters (λ = 0.05, 0.15, 0.3) on the detection of shifts in synthetic process data. The grey-shaded region represents the baseline period (n = 60 points), during which the process remains stable. The black line denotes the exponentially weighted moving average, red dashed lines indicate control limits (±2.54σ) and individual observations are plotted as black crosses. Out-of-bounds EWMA values are highlighted in red, demonstrating how smaller λ values produce smoother trends with delayed detection of shifts, whereas larger λ values respond more rapidly to deviations.
