# Supplementary material for: Predicting Affective Episodes in Bipolar Disorder Using Statistical Process Control Analysis of GPS-Based Mobility Patterns: Quantitative Study
Source: JMIR Mhealth Uhealth. 2026 Jun 22;14:e77272. doi: 10.2196/77272 (PMC13286074; doi:10.2196/77272)
Supplement: Multimedia Appendix 3 [file mhealth-v14-e77272-s003.docx]

**Supplementary Table S1: Mean (± SD) Scores of Clinician-Rated and Self-Rated Measures Across Baseline and the Eleven Mood Phases**

| Phase | YMRST Mean | BRMRS Mean | MADRS Mean | CGI-S BD Mean | Self-Rating Mean | YMRS SD | BRMRS SD | MADRS SD | CGI-S BD SD | Self-Rating SD |
| --- | --- | --- | --- | --- | --- | --- | --- | --- | --- | --- |
| Baseline | 1.06 | 0.43 | 4.65 | 1.51 | 49.25 | 1.95 | 1.23 | 4.32 | 0.87 | 12.45 |
| Euthymia | 1.06 | 0.49 | 4.26 | 1.37 | 49.89 | 1.88 | 1.13 | 5.31 | 0.70 | 10.46 |
| Depression Early Prodromal | 0.75 | 0.34 | 11.25 | 1.97 | 45.43 | 2.04 | 0.80 | 6.38 | 0.93 | 13.80 |
| Depression Late Prodroma | 0.78 | 0.28 | 11.76 | 1.86 | 42.89 | 2.03 | 0.77 | 6.91 | 0.92 | 14.77 |
| Depression First Week | 0.54 | 0.14 | 19.84 | 2.29 | 39.71 | 1.24 | 0.57 | 8.55 | 1.51 | 14.60 |
| Depression Second Week | 0.59 | 0.15 | 19.86 | 2.36 | 35.27 | 1.28 | 0.59 | 8.66 | 1.52 | 14.82 |
| Depression Ongoing Weeks | 0.61 | 0.11 | 24.90 | 2.13 | 31.14 | 1.08 | 0.32 | 10.62 | 1.68 | 16.31 |
| Mania Early Prodromal | 3.44 | 1.75 | 5.89 | 1.87 | 51.90 | 2.79 | 1.74 | 6.43 | 0.87 | 9.43 |
| Mania Late Prodromal | 3.54 | 1.96 | 5.41 | 1.96 | 50.73 | 2.79 | 1.80 | 6.37 | 0.89 | 10.63 |
| Mania First Week | 9.57 | 5.33 | 4.52 | 3.00 | 54.88 | 5.07 | 2.74 | 5.82 | 1.12 | 9.37 |
| Mania Second Week | 9.66 | 5.39 | 4.53 | 3.01 | 57.11 | 5.05 | 2.73 | 5.85 | 1.13 | 10.33 |
| Mania Ongoing Weeks | 9.98 | 5.32 | 3.92 | 3.25 | 51.24 | 4.42 | 2.22 | 4.49 | 1.03 | 10.40 |
