# Supplementary material for: Predicting Affective Episodes in Bipolar Disorder Using Statistical Process Control Analysis of GPS-Based Mobility Patterns: Quantitative Study
Source: JMIR Mhealth Uhealth. 2026 Jun 22;14:e77272. doi: 10.2196/77272 (PMC13286074; doi:10.2196/77272)
Supplement: Multimedia Appendix 4 [file mhealth-v14-e77272-s004.docx]

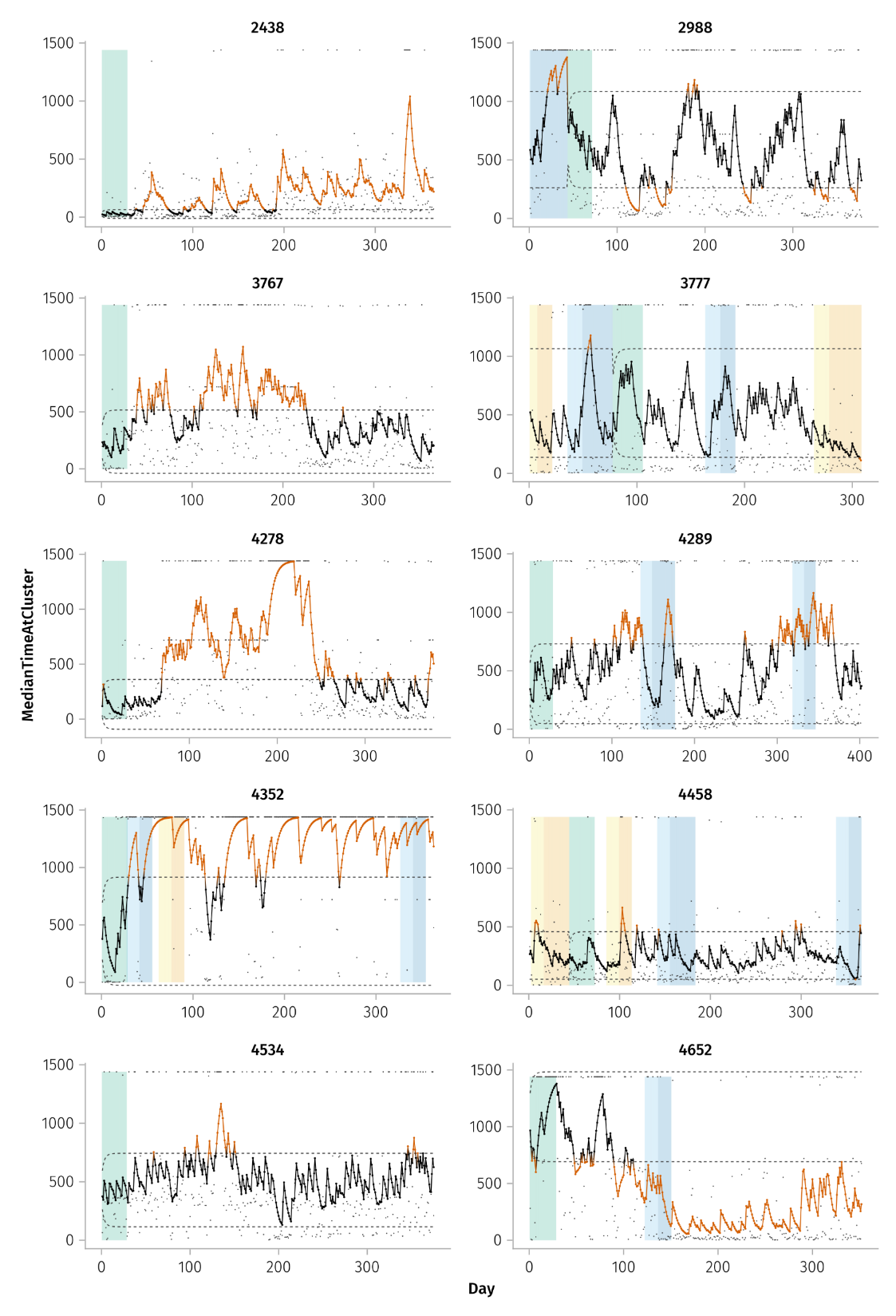

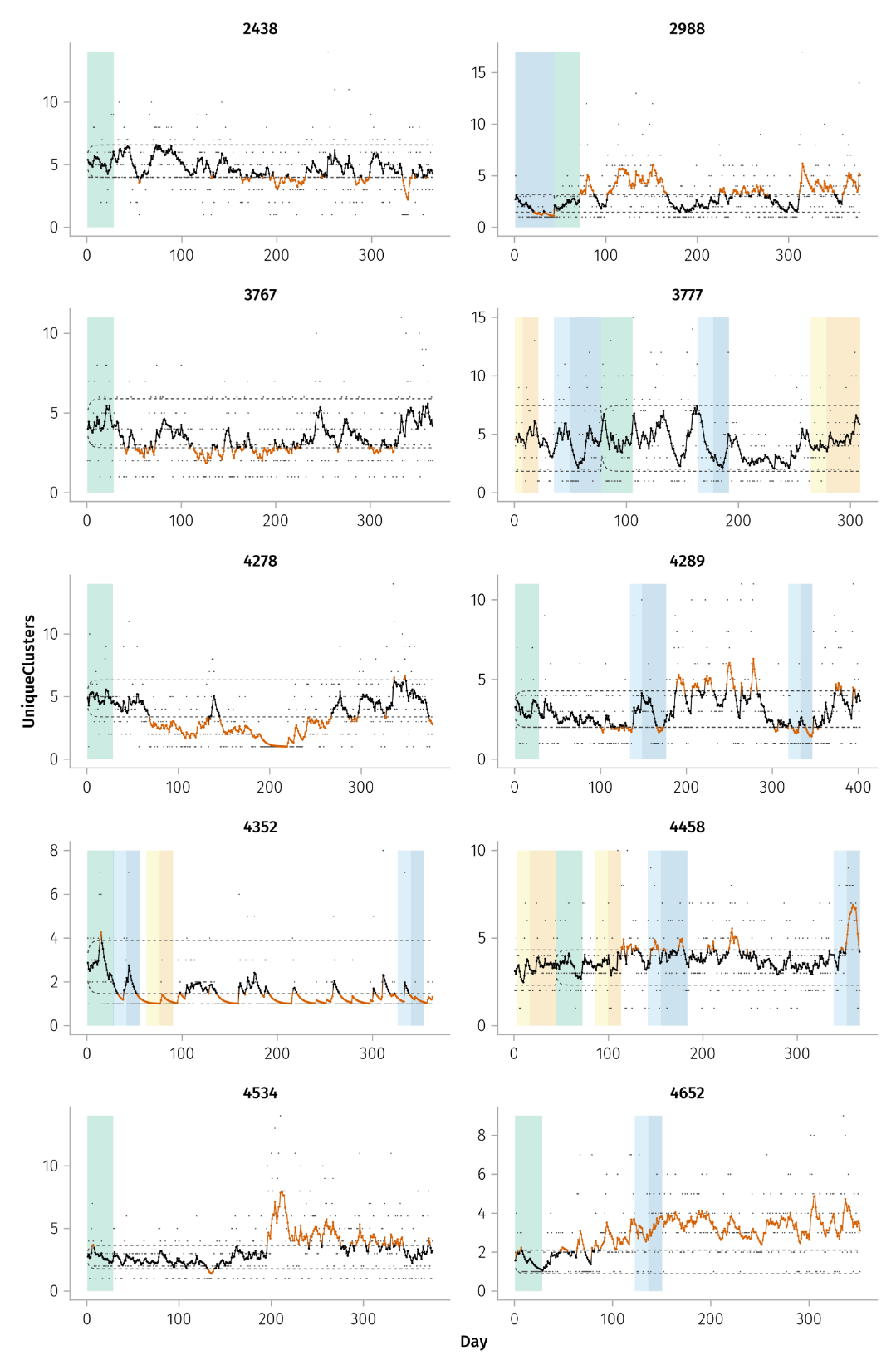

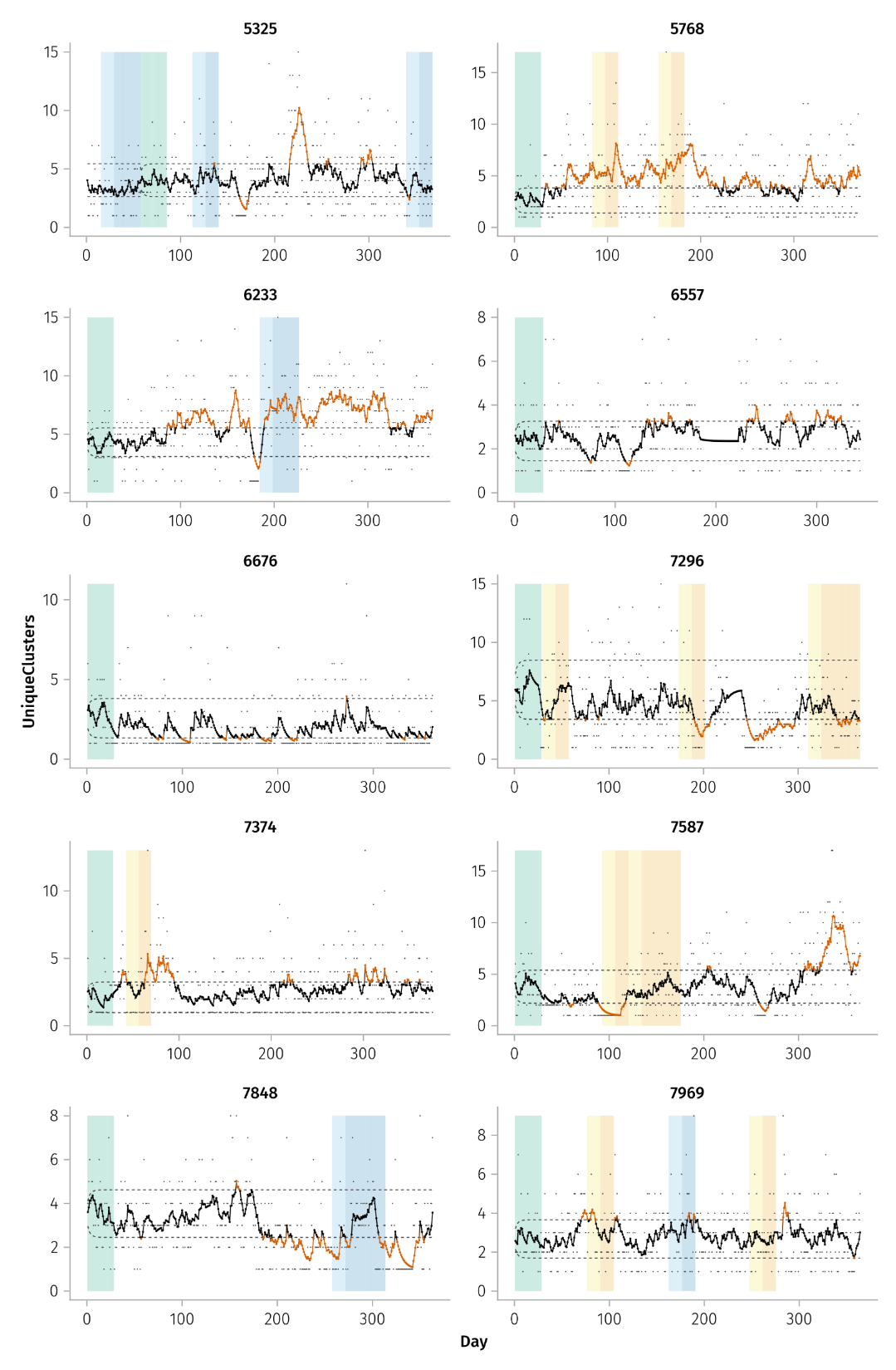

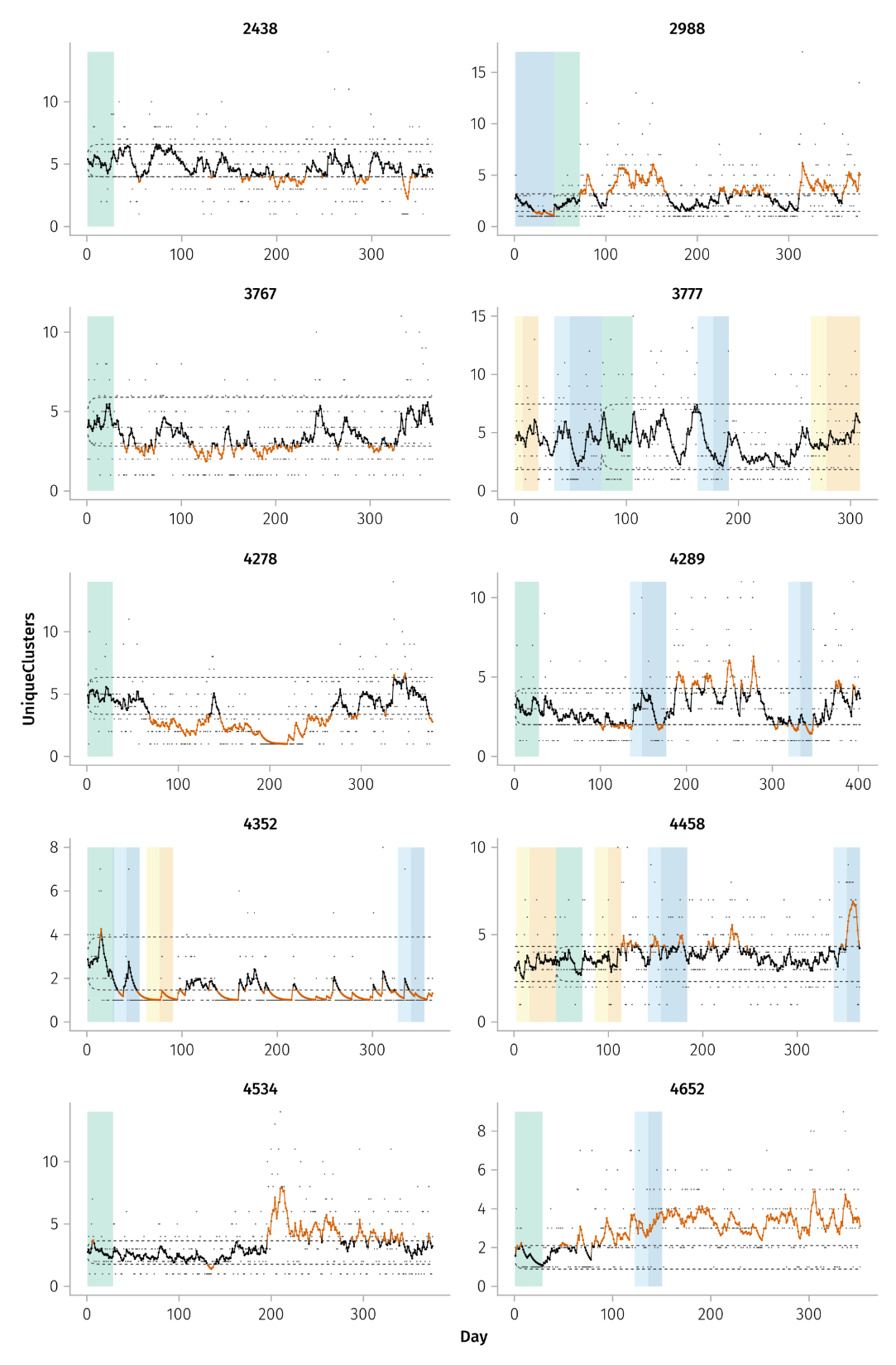

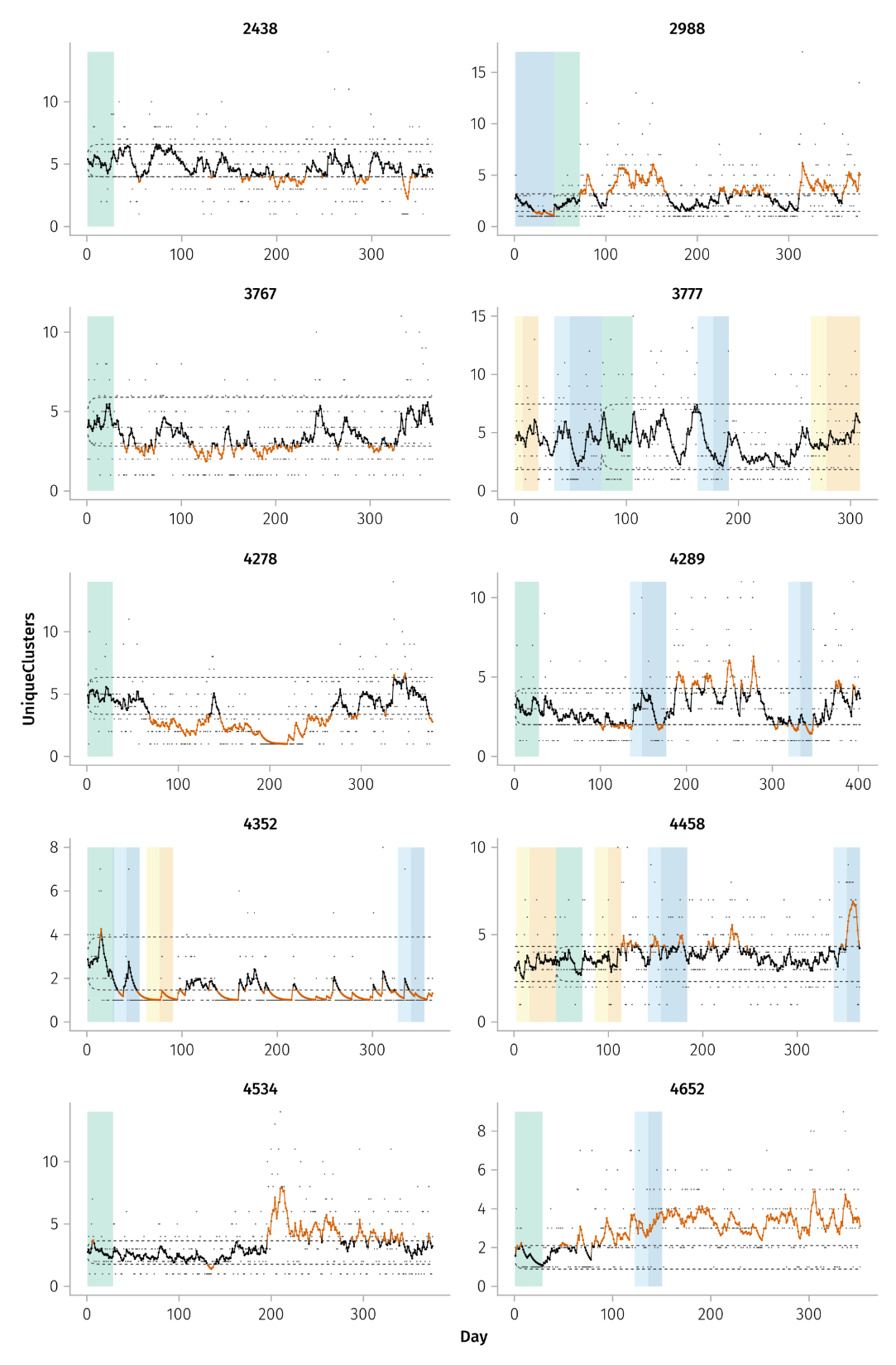

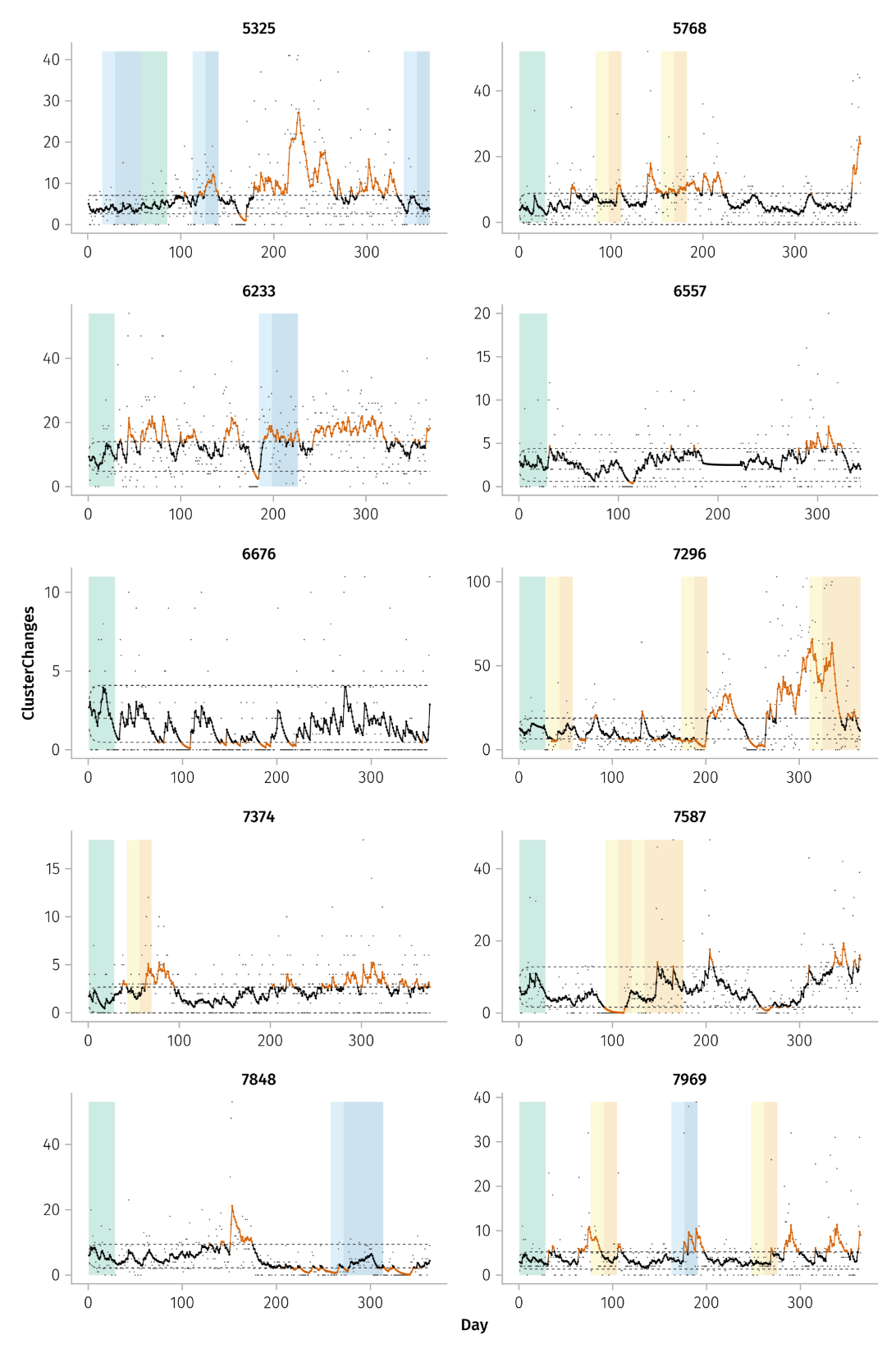

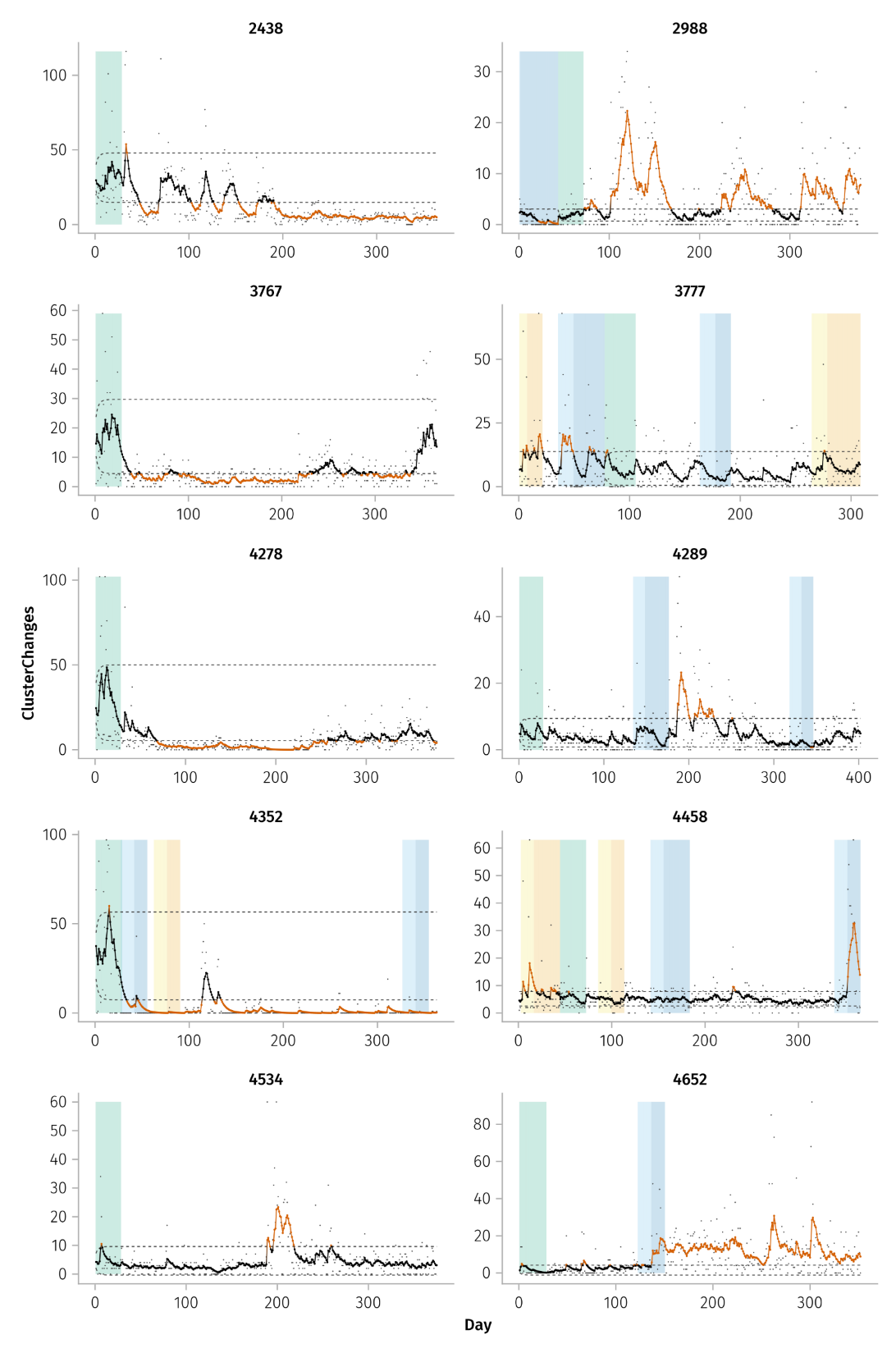

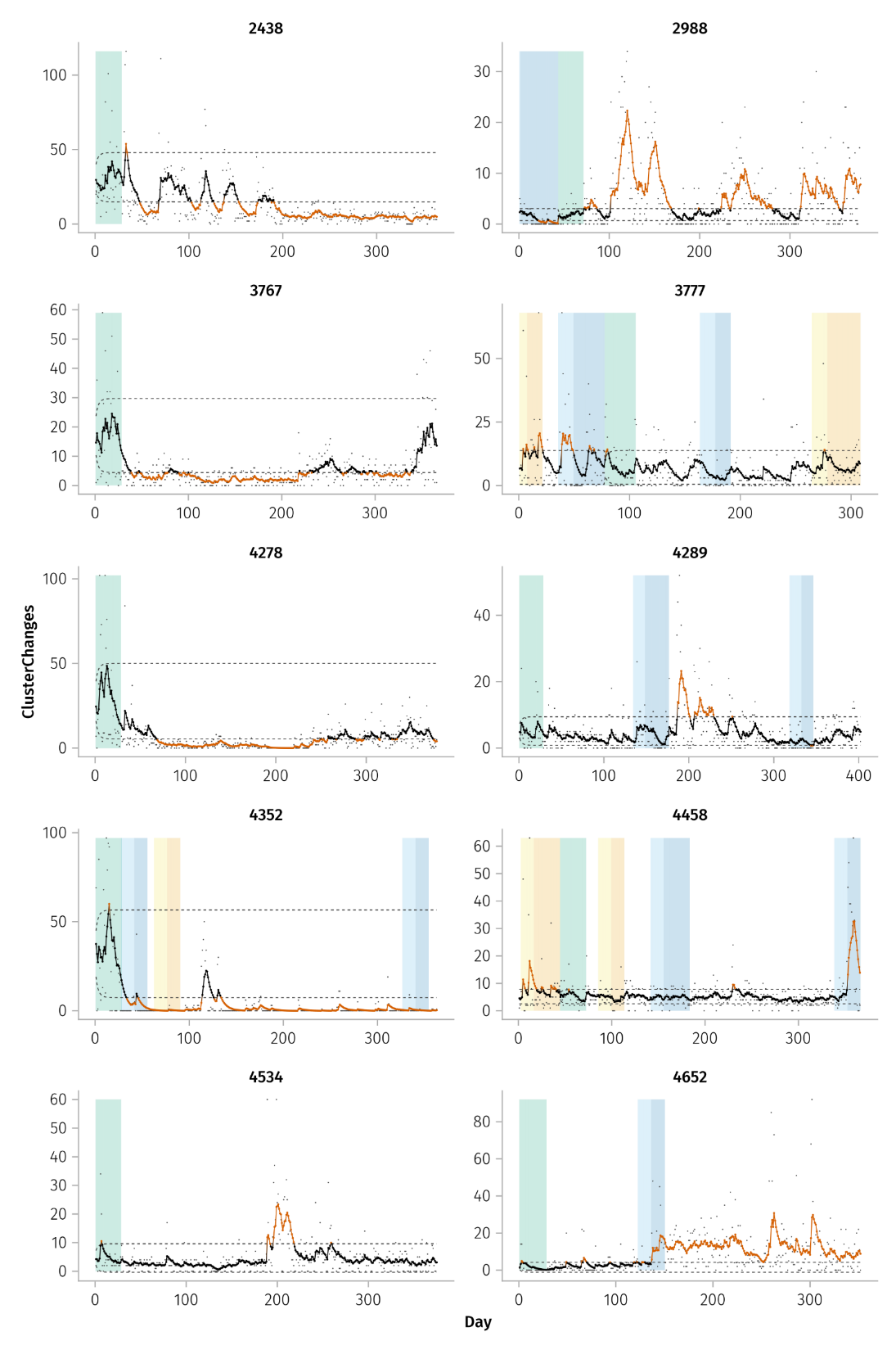

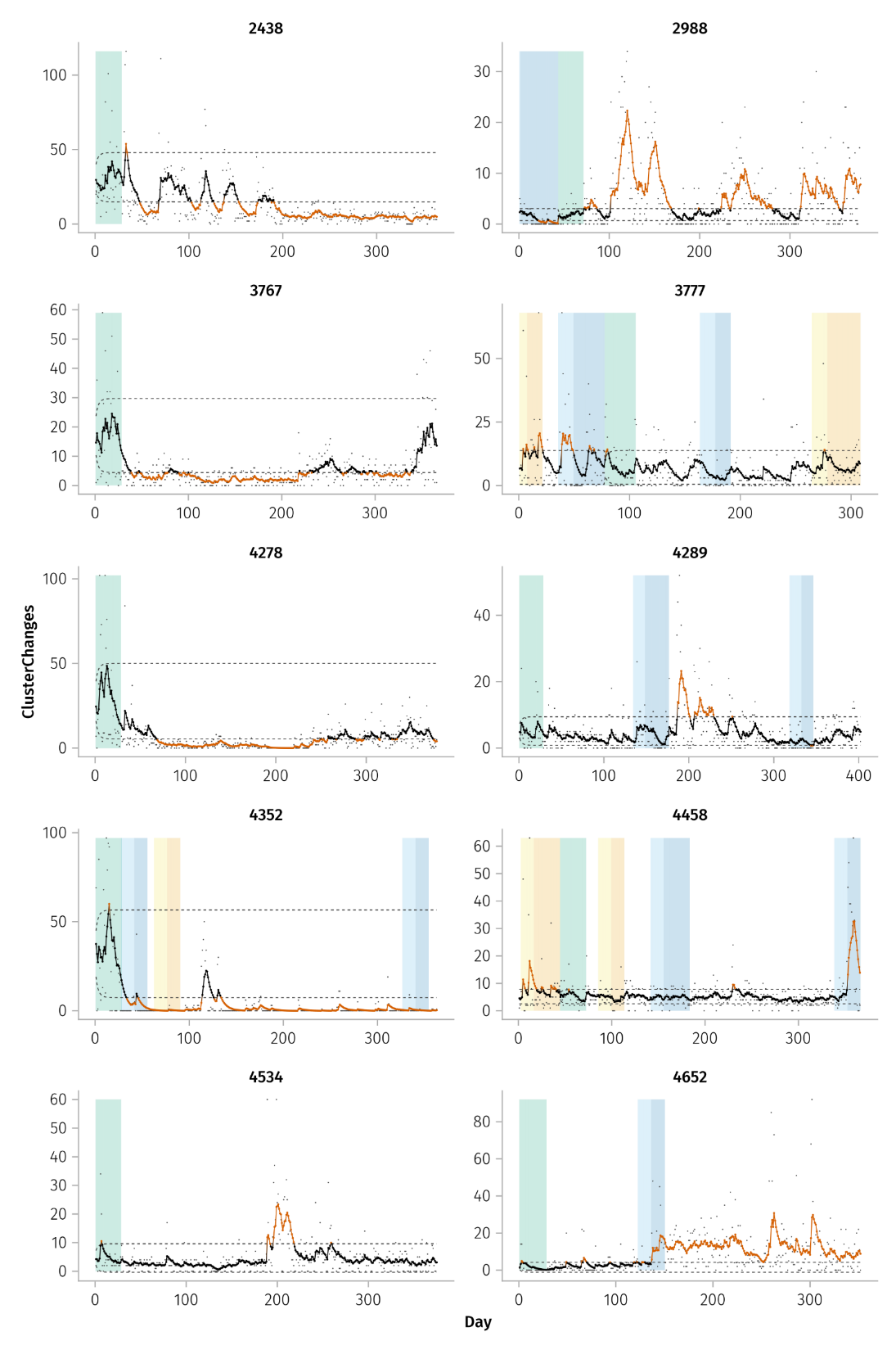

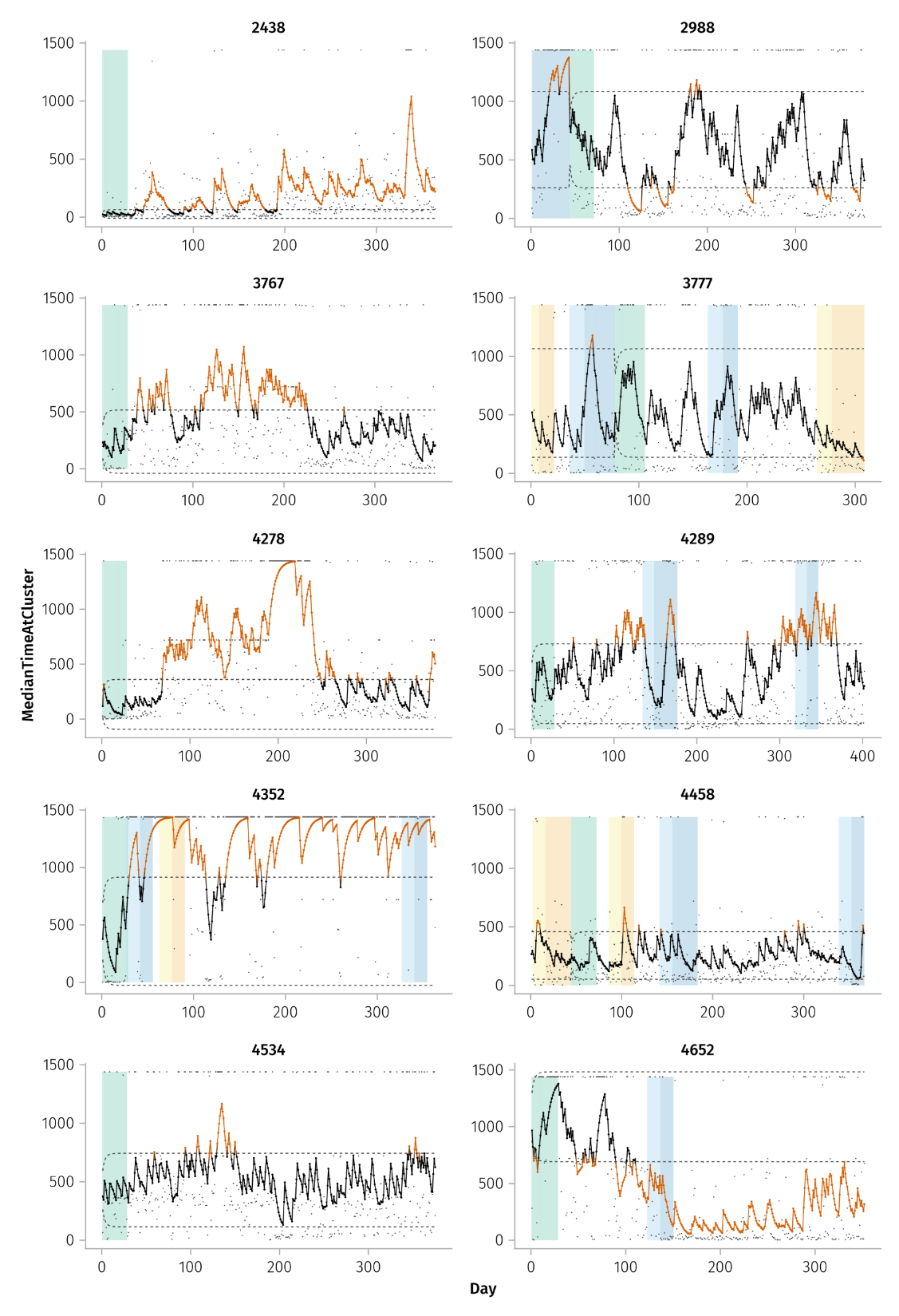

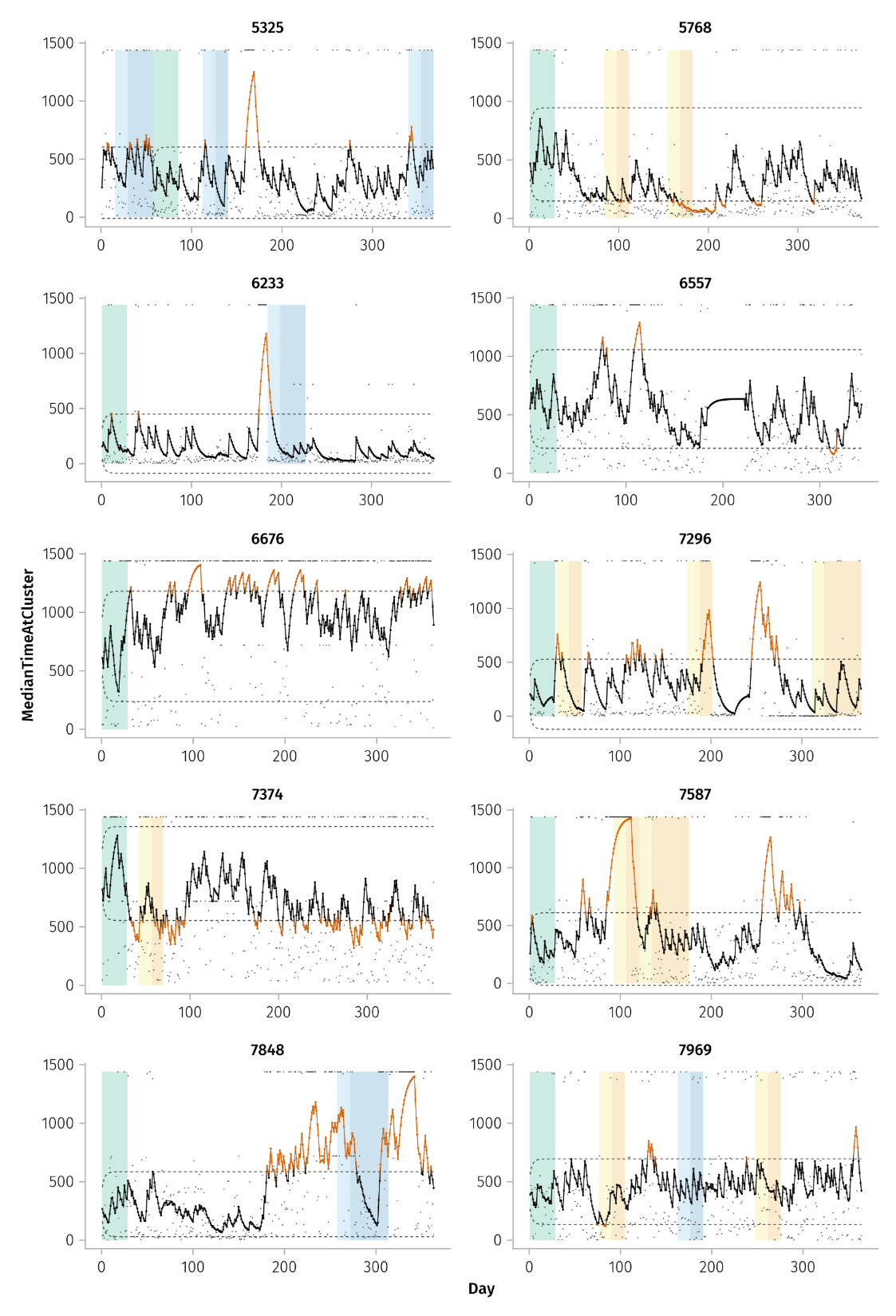

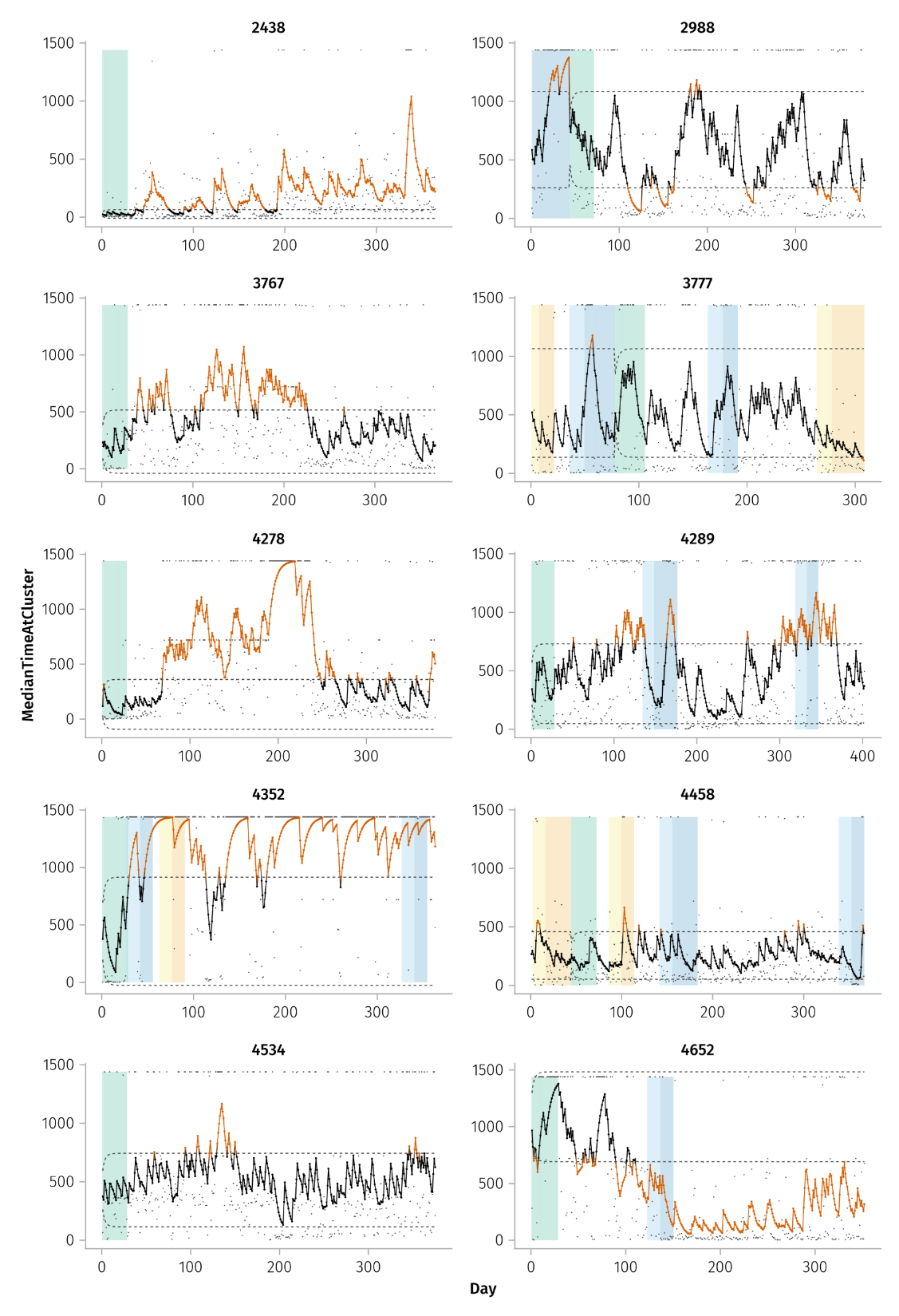


Supplementary Figure S 3: Exponentially weighted moving average (EWMA) Statistical Process Control (SPC) Chart for participants 4289 & 5768 for all remaining variables of the study. The x-axis represents the days of the study, and the y-axis shows the median time participants spent at a cluster. The baseline period, used to compute personalized upper and lower control limits, is depicted by the green area. Yellow areas indicate (hypo)manic episodes, and blue areas indicate depressive episodes. Shaded regions mark the two prodromal weeks (early and late prodromal). Black dots represent daily raw values, and the black line depicts the EWMA. The line turns red when the EWMA exceeds the control limits.
