# Supplementary material for: Predicting Affective Episodes in Bipolar Disorder Using Statistical Process Control Analysis of GPS-Based Mobility Patterns: Quantitative Study
Source: JMIR Mhealth Uhealth. 2026 Jun 22;14:e77272. doi: 10.2196/77272 (PMC13286074; doi:10.2196/77272)
Supplement: Multimedia Appendix 5 [file mhealth-v14-e77272-s005.docx]

**Supplementary Table S2: Results of the optimization procedure of Lambda and L**

| **Variable** | **Lambda** | **L** | **Loss** | **Correlation** | **Accuracy** | **Sensitivity** | **Specificity** | **Precision** | **F1-Score** | **Balanced Accuracy** | **Cohen’s Kappa** |
| --- | --- | --- | --- | --- | --- | --- | --- | --- | --- | --- | --- |
| UniqueClusters | 0.01 | 0.7 | Correlation | 0.0639 | 0.253 | 0.925 | 0.134 | 0.159 | 0.271 | 0.530 | 0.020 |
| UniqueClusters | 0.96 | 5.0 | Accuracy | 0.0021 | 0.841 | 0.013 | 0.988 | 0.157 | 0.023 | 0.500 | 0.001 |
| UniqueClusters | 0.01 | 0.6 | F2-Score | 0.0607 | 0.241 | 0.935 | 0.118 | 0.158 | 0.270 | 0.527 | 0.018 |
| UniqueClusters | 0.01 | 0.7 | F1-Score | 0.0639 | 0.253 | 0.925 | 0.134 | 0.159 | 0.271 | 0.530 | 0.020 |
| UniqueClusters | 0.01 | 0.7 | Balanced Accuracy | 0.0639 | 0.253 | 0.925 | 0.134 | 0.159 | 0.271 | 0.530 | 0.020 |
| UniqueClusters | 0.02 | 4.9 | Cohen’s Kappa | 0.0350 | 0.602 | 0.412 | 0.635 | 0.166 | 0.237 | 0.524 | 0.029 |
| UniqueClusters | 0.15 | 2.54 | - | -0.0192 | 0.613 | 0.308 | 0.666 | 0.140 | 0.193 | 0.487 | -0.017 |
| ClusterChanges | 0.01 | 0.9 | Correlation | 0.0597 | 0.272 | 0.899 | 0.161 | 0.159 | 0.271 | 0.530 | 0.021 |
| ClusterChanges | 0.99 | 5.0 | Accuracy | -0.0144 | 0.835 | 0.014 | 0.981 | 0.113 | 0.025 | 0.497 | -0.009 |
| ClusterChanges | 0.01 | 0.1 | F2-Score | 0.0267 | 0.166 | 0.989 | 0.021 | 0.152 | 0.263 | 0.505 | 0.003 |
| ClusterChanges | 0.01 | 0.9 | F1-Score | 0.0597 | 0.272 | 0.899 | 0.161 | 0.159 | 0.271 | 0.530 | 0.021 |
| ClusterChanges | 0.01 | 1.0 | Cohen’s Kappa | 0.0571 | 0.281 | 0.885 | 0.175 | 0.159 | 0.270 | 0.530 | 0.021 |
| ClusterChanges | 0.15 | 2.54 | - | -0.0473 | 0.581 | 0.307 | 0.629 | 0.128 | 0.180 | 0.468 | -0.040 |
| MedianTimeAtCluster | 0.02 | 0.7 | Correlation | 0.0725 | 0.288 | 0.895 | 0.181 | 0.162 | 0.274 | 0.538 | 0.027 |
| MedianTimeAtCluster | 1.0 | 5.0 | Accuracy | 0.0217 | 0.847 | 0.010 | 0.995 | 0.250 | 0.019 | 0.502 | 0.008 |
| MedianTimeAtCluster | 0.02 | 0.4 | F2-Score | 0.0590 | 0.232 | 0.943 | 0.107 | 0.157 | 0.270 | 0.525 | 0.016 |
| MedianTimeAtCluster | 0.02 | 0.7 | F1-Score | 0.0725 | 0.288 | 0.895 | 0.181 | 0.162 | 0.274 | 0.538 | 0.027 |
| MedianTimeAtCluster | 1.0 | 0.7 | Cohen’s Kappa | 0.0479 | 0.503 | 0.577 | 0.490 | 0.167 | 0.259 | 0.534 | 0.033 |
| MedianTimeAtCluster | 0.15 | 2.54 | - | 0.0047 | 0.637 | 0.312 | 0.694 | 0.153 | 0.205 | 0.503 | 0.004 |
